# Supplementary material for: Sparse Labeling PELDOR Spectroscopy on Multimeric Mechanosensitive Membrane Channels
Source: Biophys J. 2017 Nov 7;113(9):1968–78. doi: 10.1016/j.bpj.2017.09.005 (PMC5685675; doi:10.1016/j.bpj.2017.09.005)
Supplement: Document S1. Supporting Materials and Methods and Figs. S1–S37 [file mmc1.pdf]

Biophysical Journal, Volume *113*

Supplemental Information

Sparse Labeling PELDOR Spectroscopy on Multimeric Mechanosensi-tive Membrane Channels

Katrin Ackermann, Christos Pliotas, Silvia Valera, James H. Naismith, and Bela E. Bode

Table of Contents

Chapter 1: Modeling results page 3
Chapter 2: Additional PELDOR data page 7
2.1) MscS S196R1 (cytosolic mutant)
2.2) MscL V120R1 (cytosolic mutant)
2.3) MscS D67R1 (transmembrane mutant)
2.4) MscL M94R1 (transmembrane mutant)
Chapter 3: Dipolar dephasing page 21
Chapter 4: Peak intensities page 24
Chapter 5: Supporting references page 25

Chapter 1: Modeling results

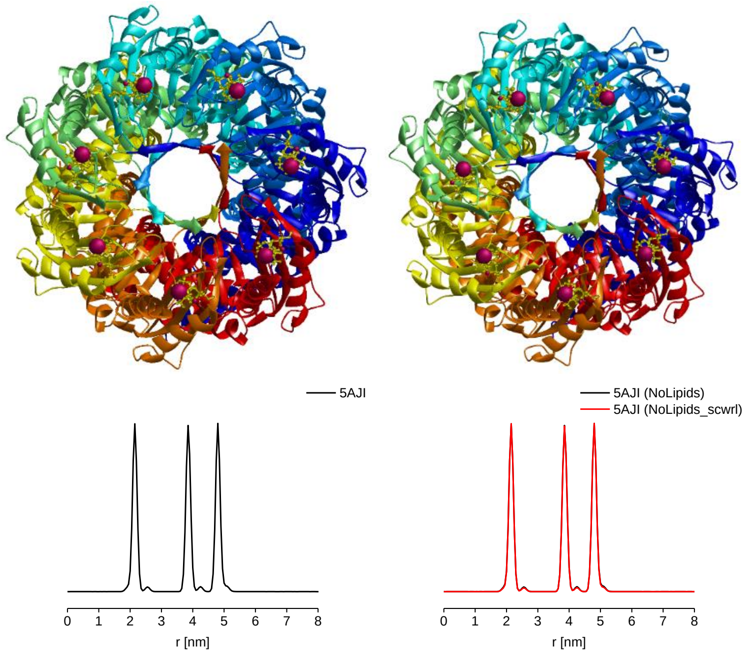

Figure S1: Modeling results for MscS S196R1 based on crystal structure pdb 5aji generated using MMM2015 (1,2). Top: Models with spin-label attached (left: pdb 5aji; right: pdb 5aji with lipids removed and side-chains repacked). Monomers are individually colored. Bottom: Distance distributions comparing the original pdb with the lipids removed and the side-chains repacked (scwrl). Side-chain repacking was not possible with the lipids still present. Virtually no differences between models and distance distributions can be observed.

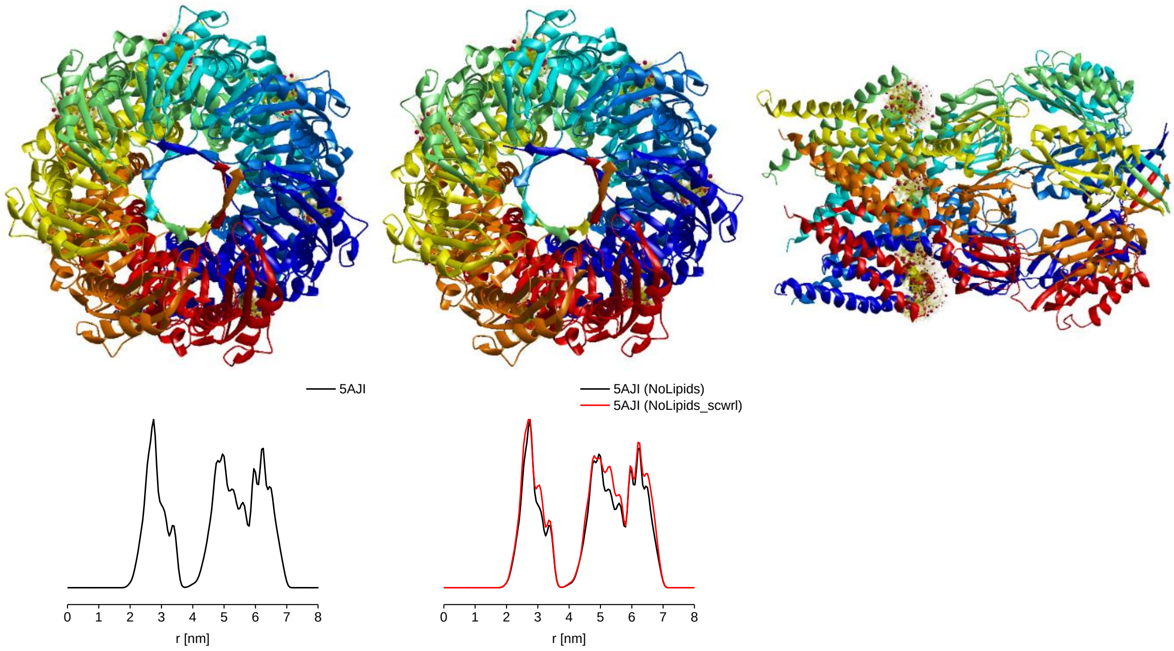

Figure S2: Modeling results for MscS D67R1 based on crystal structure pdb 5aji generated using MMM2015 (1,2). The spin-labelled residue at position 67 was mutated to a cysteine before the site-scan. Top: Models with spin-label attached (left: pdb 5aji; middle (top view) and right (side view): pdb 5aji with lipids removed and side-chains repacked). Monomers are individually colored. Bottom: Distance distributions comparing the original pdb with the lipids removed and the side-chains repacked (scwrl). Side-chain repacking was not possible with the lipids still present. Only minor differences between the distance distributions can be observed.

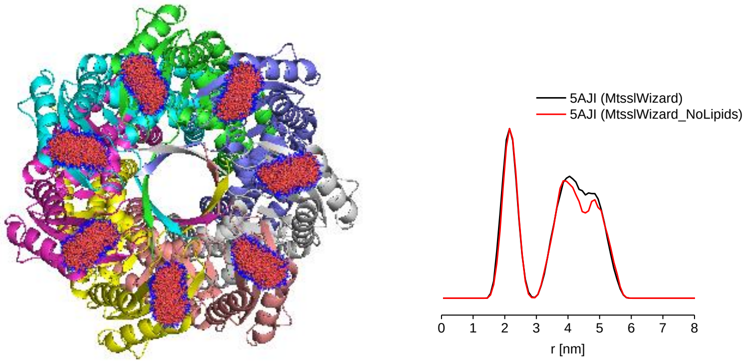

Figure S3: Modeling results for MscS S196R1 based on crystal structure pdb 5aji generated using MtsslWizard (3). Left: model with spin-labels attached; monomers are individually colored. Right: Distance distributions with and without lipids obtained for painstaking search at "loose" van-der-Waals cut-off settings (2.5 angstroms cut-off and 5 clashes allowed) as with the "tight" setting (3.4 angstroms cut-off and no clashes allowed) no rotamers were found. This "loose" setting might be attributable to the broader distribution compared to MMM.

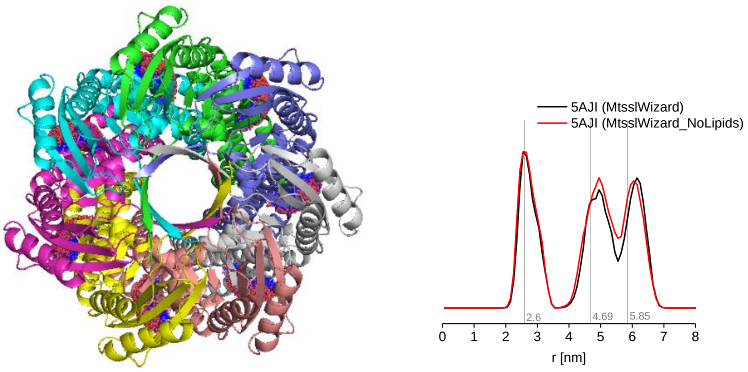

Figure S4: Modeling results for MscS D67R1 based on crystal structure pdb 5aji generated using MtsslWizard (3). Left: model with spin-labels attached; monomers are individually colored. Right: Distance distributions with and without lipids obtained for painstaking search at "tight" van-der-Waals cut-off settings. Only minor differences were observed. Vertical lines indicate the direct distance measurement from the position of the radical (approximated to be localized on the oxygen atom of the nitroxyl group) in the crystal structure in PyMOL; exact measurements are given in nm.

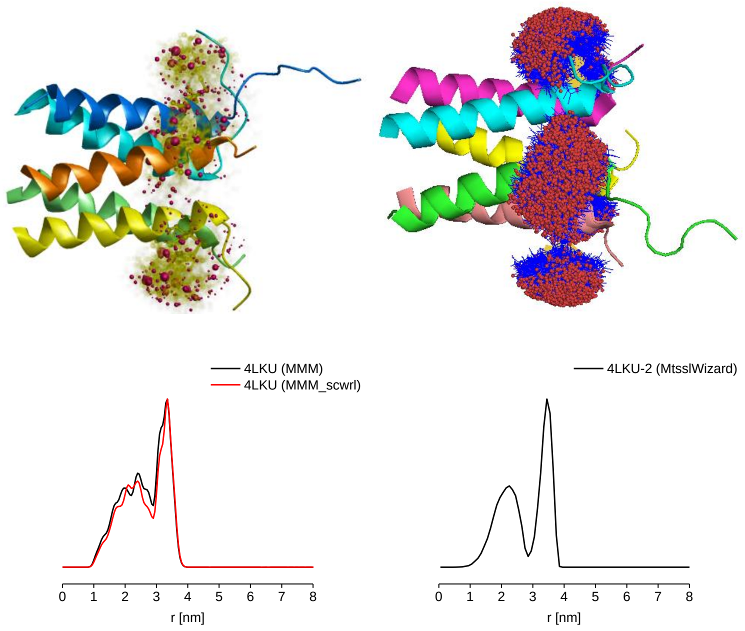

Figure S5: Top: Models of the cytosolic domain of *E.coli* MscL V120R1 based on crystal structure pdb 4lku generated using MMM2015.1 (left, side-chains re-packed) or MtsslWizard (right). Monomers are individually colored. Bottom: Corresponding distance distributions, in the case of MMM (left) with (scwrl) and without re-packing of side-chains. Only minor differences can be observed.

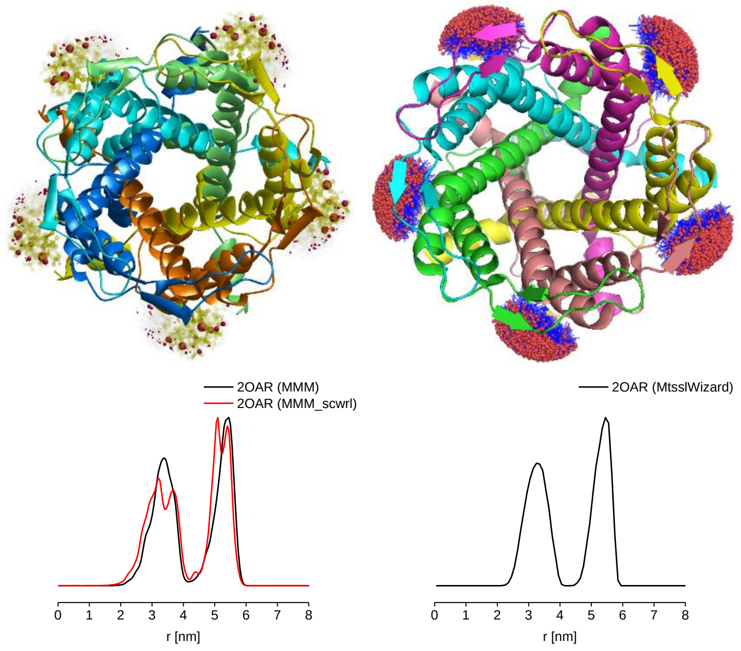

Figure S6: Top: Models of *M. tuberculosis* MscL F88R1 (corresponding to residue M94 in *E. coli* (4)) based on crystal structure pdb 2oar generated using MMM2015.1 (left, side-chains re-packed) or MtsslWizard (right). Monomers are individually colored. Bottom: Corresponding distance distributions, in the case of MMM (left) with (scwrl) and without re-packing of side-chains. Only minor differences can be observed.

Chapter 2: Additional PELDOR data

With the exception of Figure S30, all figures in this chapter are arranged in a similar way. Raw PELDOR data are shown on the top left. Background-corrected data with fit are shown on the top right. Corresponding distance distributions can be found on the bottom left, whereby the gray vertical line indicates the cut-off distance (at r /nm = (t /µs × 52)$^{1/3}$). The 95% confidence interval (±2σ) of the distance distributions obtained by statistical analysis is shown on the bottom right overlaid with the structural model in light gray. Fits and distance distributions are shown both, without (black) and with (red) power-scaling. All distance distributions are shown with color bars representing respective reliability ranges as described in detail in the DeerAnalysis manual (5) (green: shape reliable; yellow: mean and width reliable; orange: mean reliable; red: no quantification possible).

2.1) MscS S196R1 (cytosolic mutant)

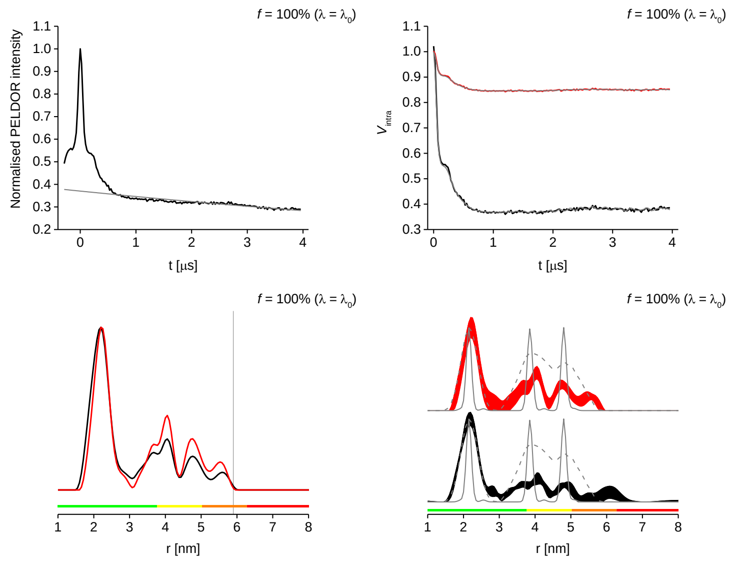

Figure S7: Q-band PELDOR data for MscS S196R1 at 100% labeling, full λ. See page 6 for details.

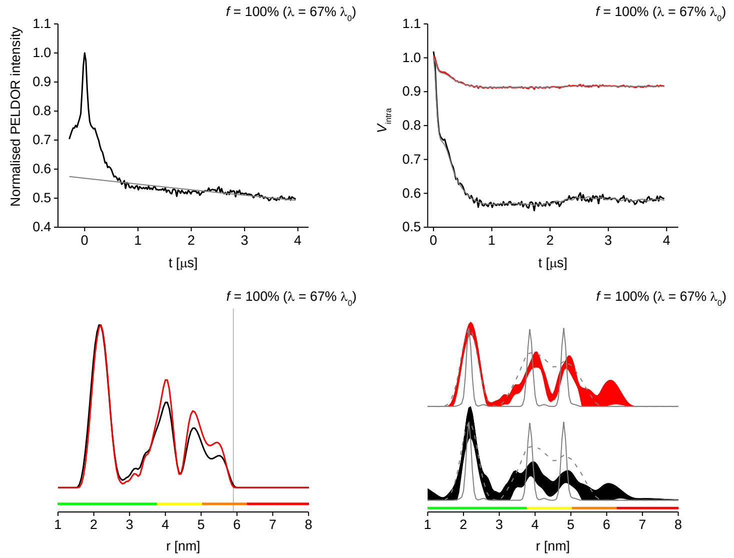

Figure S8: Q-band PELDOR data for MscS S196R1 at 100% labeling, λ = 67% λ_0. See page 6 for details.

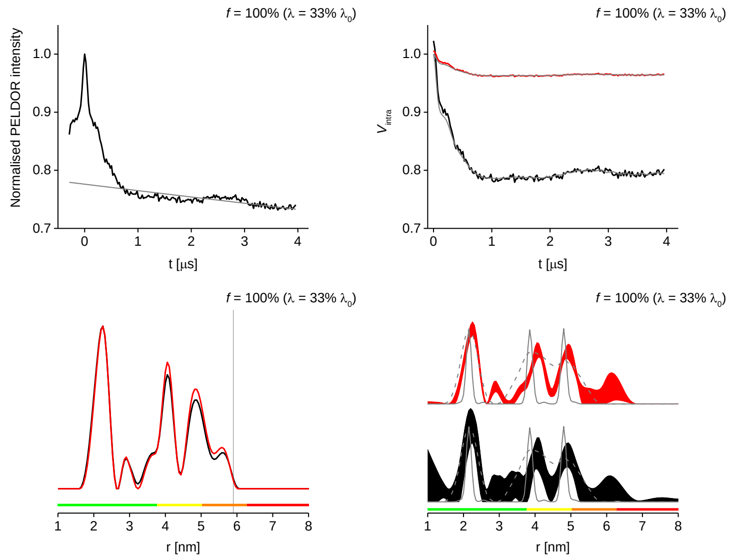

Figure S9: Q-band PELDOR data for MscS S196R1 at 100% labeling, λ = 33% λ_0. See page 6 for details.

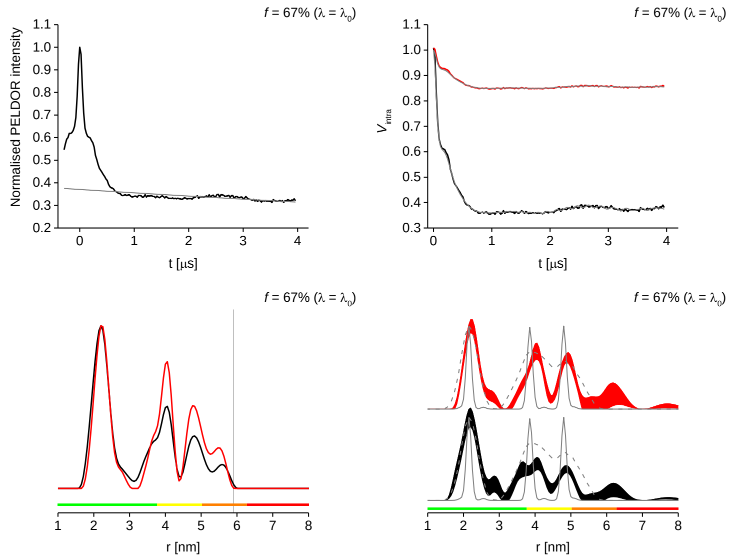

Figure S10: Q-band PELDOR data for MscS S196R1 at 67% labeling, full λ. See page 6 for details.

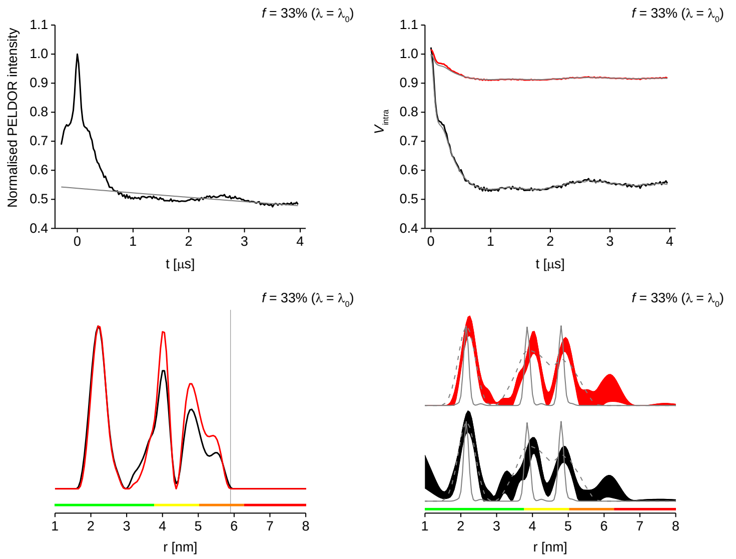

Figure S11: Q-band PELDOR data for MscS S196R1 at 33% labeling, full λ. See page 6 for details.

2.2) MscL V120R1 (cytosolic mutant)

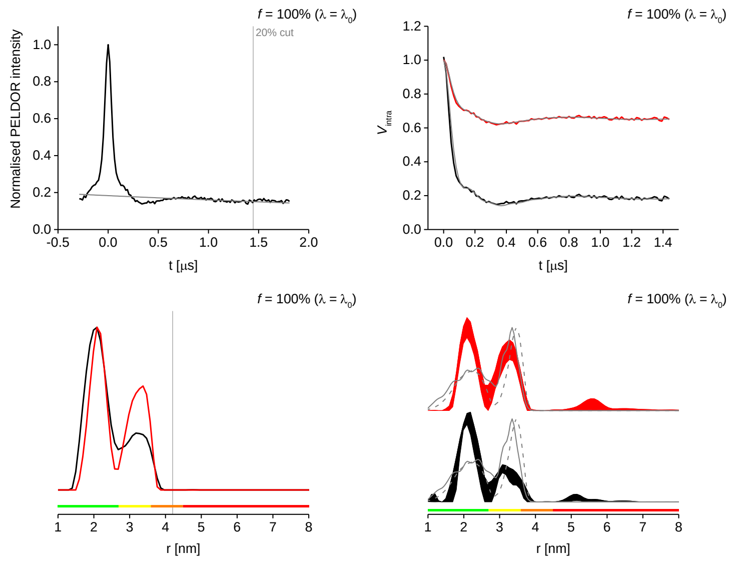

Figure S12: X-band PELDOR data for MscL V120R1, fully labeled, full λ. See page 6 for details.

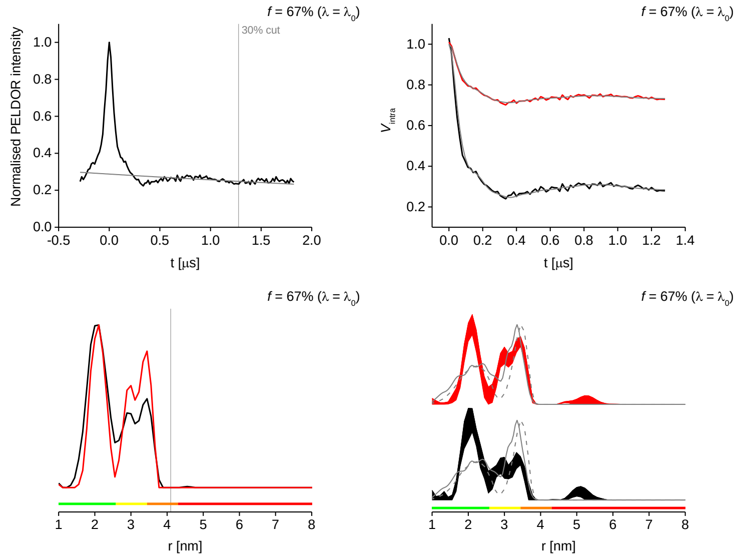

Figure S13: X-band PELDOR data for MscL V120R1 at 67% labeling, full λ. See page 6 for details.

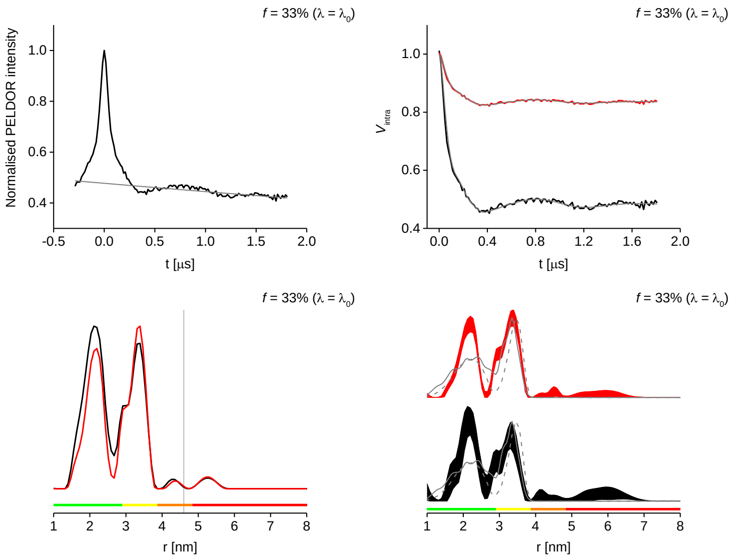

Figure S14: X-band PELDOR data for MscL V120R1 at 33% labeling, full λ. See page 6 for details.

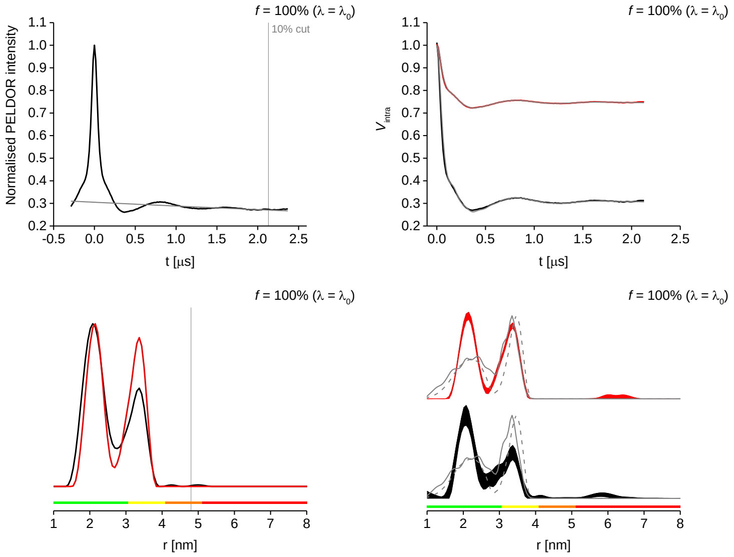

Figure S15: Q-band PELDOR data for MscL V120R1 at 100% labeling, full λ. See page 6 for details.

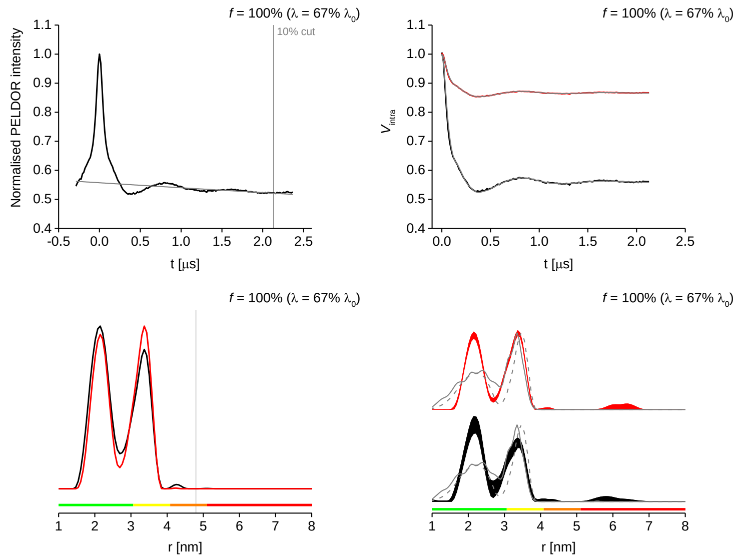

Figure S16: Q-band PELDOR data for MscL V120R1 at 100% labeling, λ = 67% λ_0. See page 6 for details.

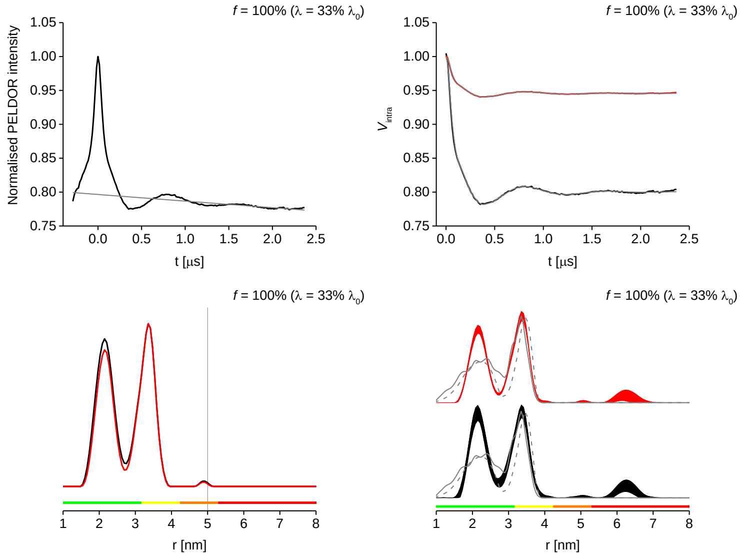

Figure S17: Q-band PELDOR data for MscL V120R1 at 100% labeling, λ = 33% λ_0. See page 6 for details.

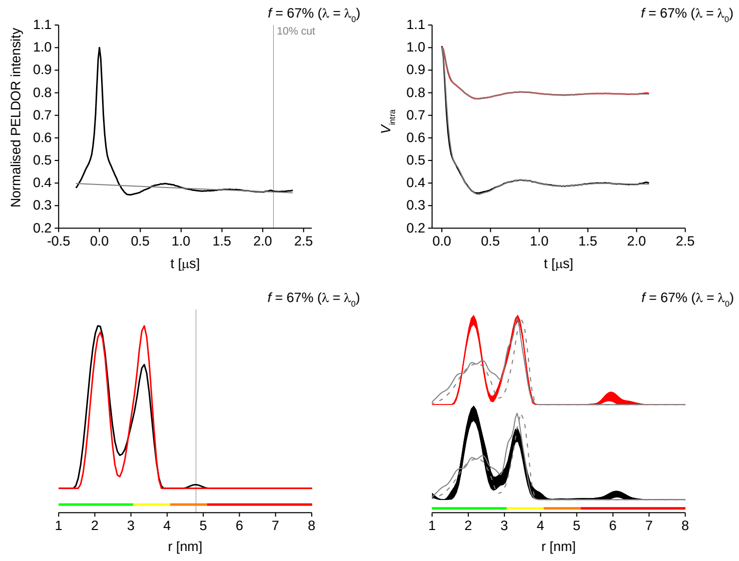

Figure S18: Q-band PELDOR data for MscL V120R1 at 67% labeling, full λ. See page 6 for details.

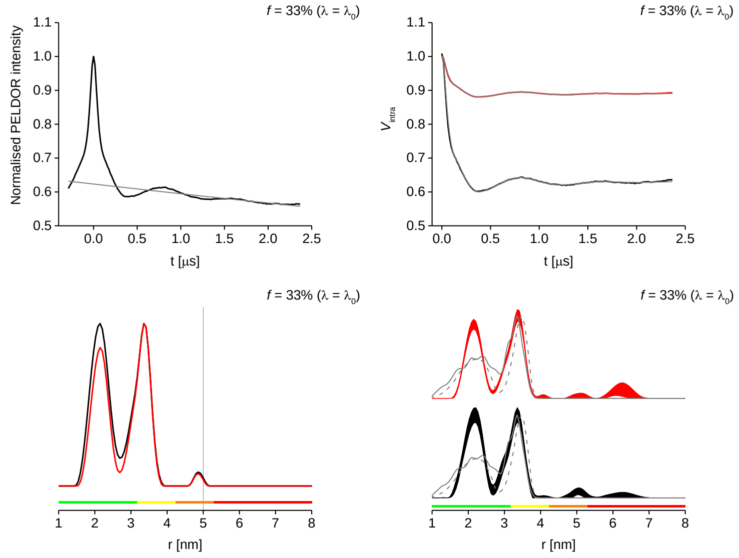

Figure S19: Q-band PELDOR data for MscL V120R1 at 33% labeling, full λ. See page 6 for details.

2.3) MscS D67R1 (transmembrane mutant)

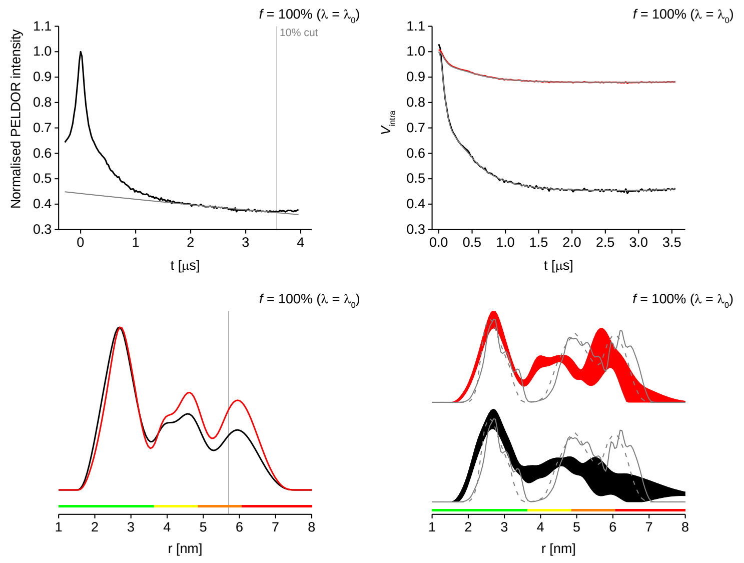

Figure S20: Q-band PELDOR data for MscS D67R1 at 100% labeling, full λ. See page 6 for details.

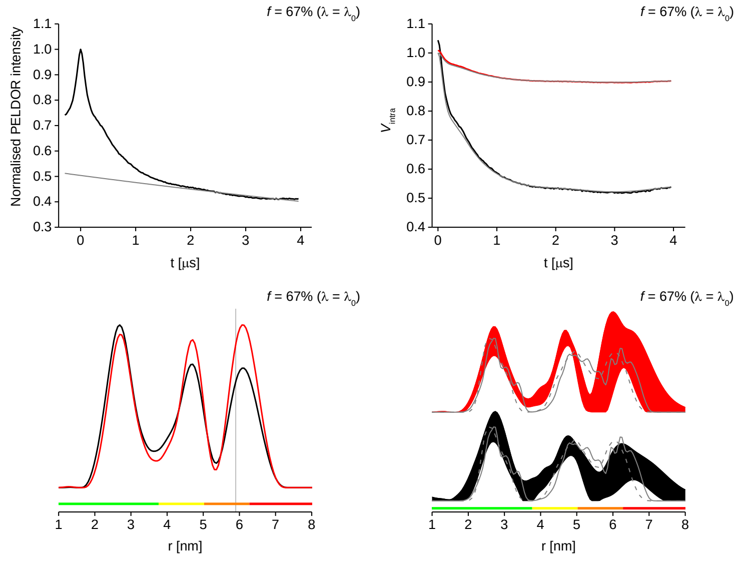

Figure S21: Q-band PELDOR data for MscS D67R1 at 67% labeling, full λ. See page 6 for details.

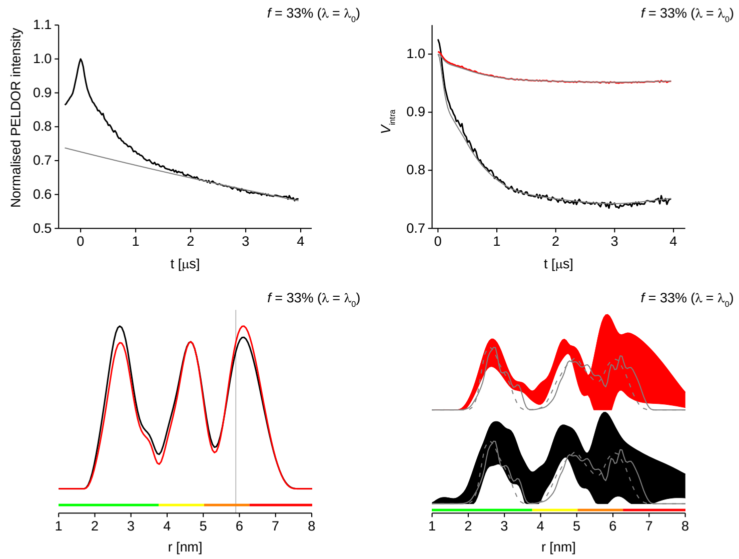

Figure S22: Q-band PELDOR data for MscS D67R1 at 33% labeling, full λ. See page 6 for details.

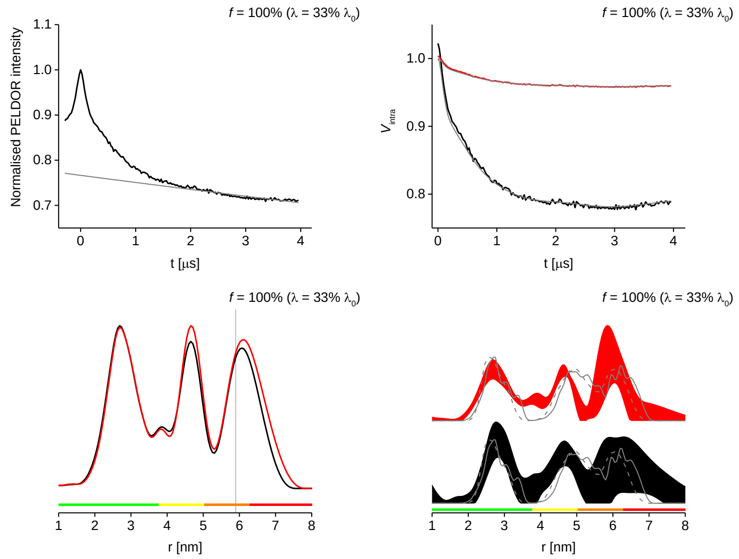

Figure S23: Q-band PELDOR data for MscS D67R1 at 100% labeling, λ = 33% λ_0. See page 6 for details.

2.4) MscL M94R1 (transmembrane mutant)

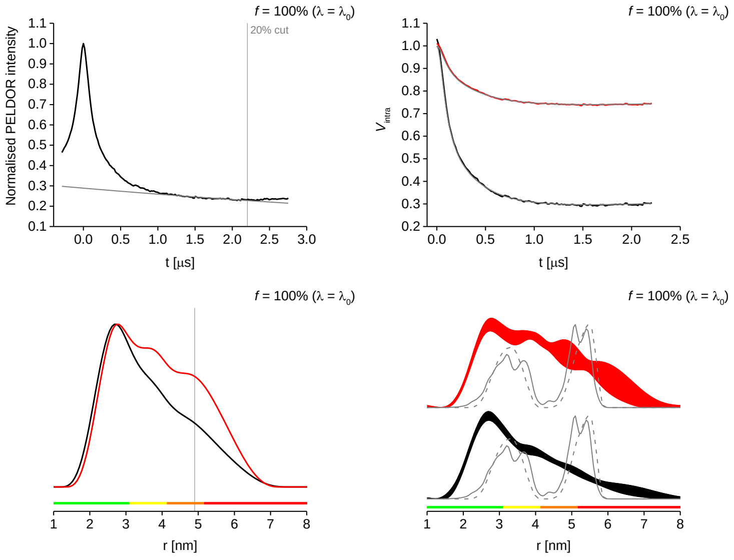

Figure S24: Q-band PELDOR data for MscL M94R1 at 100% labeling, full λ. See page 6 for details.

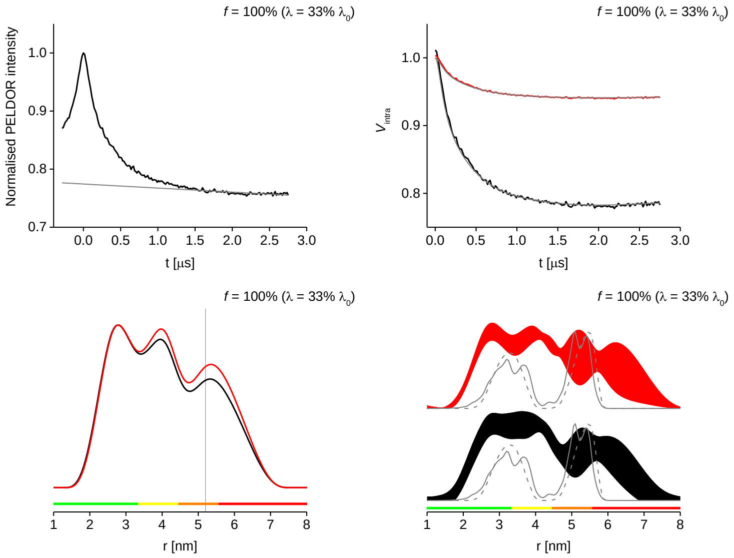

Figure S25: Q-band PELDOR data for MscL M94R1 at 100% labeling, λ = 33% λ_0. See page 6 for details.

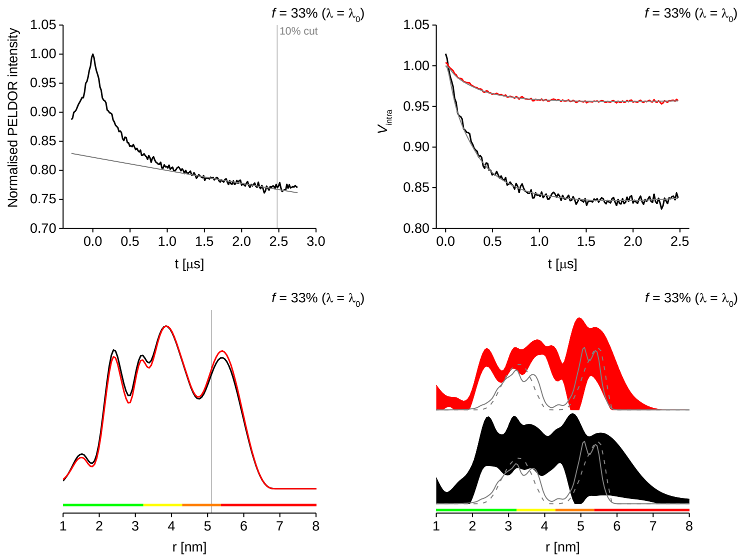

Figure S26: Q-band PELDOR data for MscL M94R1 at 33% labeling, full λ. See page 6 for details.

MscL M94 R1 – second data set with longer *t* (4 μs) used only for scaled fits (see below)

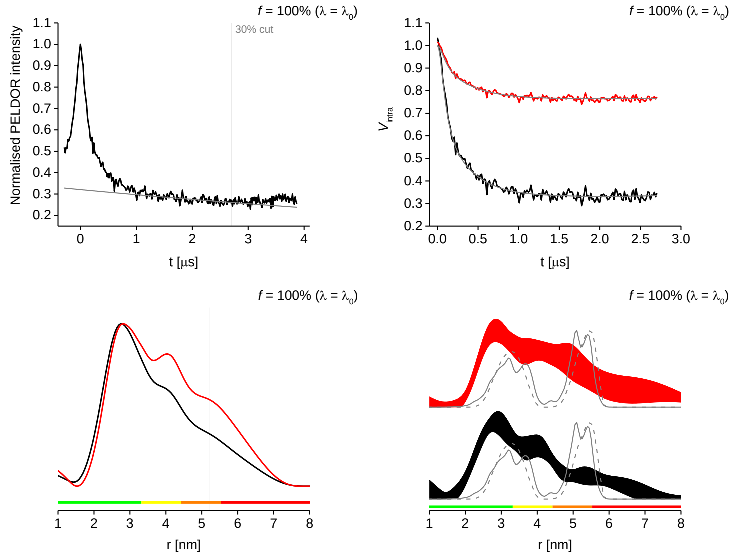

Figure S27: Q-band PELDOR data for MscL M94R1 at 100% labeling, full λ. See page 6 for details.

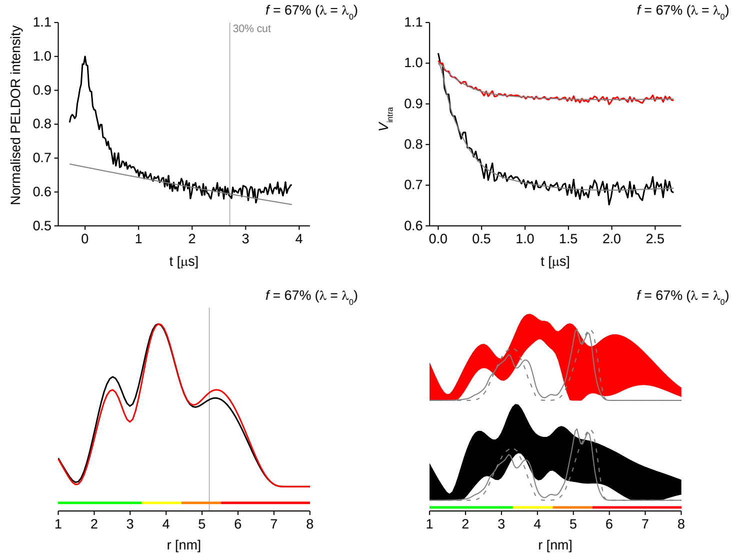

Figure S28: Q-band PELDOR data for MscL M94R1 at 67% labeling, full λ. See page 6 for details.

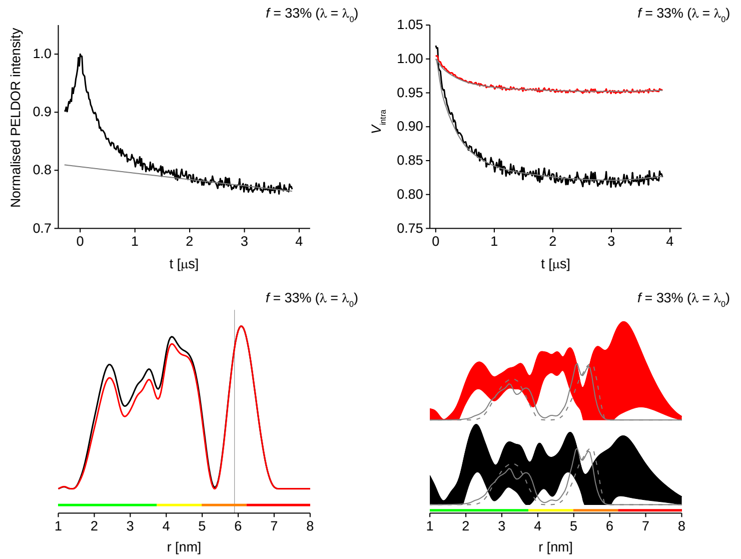

Figure S29: Q-band PELDOR data for MscL M94R1 at 33% labeling, full λ. See page 6 for details.

MscL M94 R1 – scaled fits

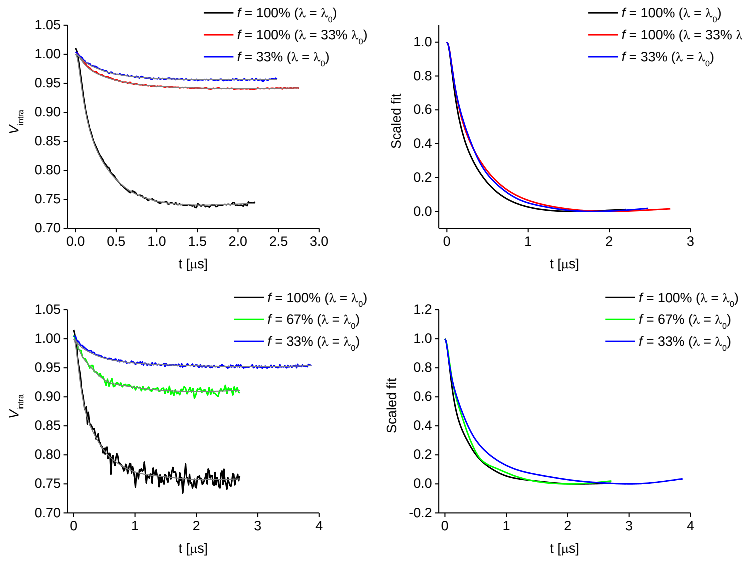

Figure S30: Background-corrected traces (left) and scaled fits (right) for MscL M94R1. Data shown in the top row corresponds to the PELDOR distance distributions shown in the manuscript; detailed PELDOR data for the bottom row are given above. The dipolar evolution functions illustrate the slightly reduced decay of the intramolecular signal observable as a 'reduced curvature' with reduced labeling and λ.

Chapter 3: Dipolar dephasing

The contribution of dipolar dephasing to the signal decay was tested by means of Hahn echo decays varying the flip-angle of the second pulse from π to π/5. A pure instantaneous diffusion effect would be expected to follow Eq. S1:

$$\frac{V(2\tau)}{V(0)} = exp\left[-\frac{2\pi}{9\times\sqrt{3}}\frac{\mu_0 g^2 \beta_e^2}{\hbar} C\tau \int sin^2\left(\frac{\beta(\Omega_S)}{2}\right) f(\Omega_S) d\Omega_S\right] \quad \text{Equation S1}$$

g is the proportionality factor (g-value), $\hbar$ the Planck constant divided by 2π, μ_0 the permeability of the vacuum, τ the interpulse delay, C the volume concentration of the spins, $\beta(\Omega_S)$ the flip angle of the second pulse as a function of resonance offset, Ω_S the resonance offset and $f(\Omega_S)$ the EPR lineshape function.

According to Eq. S1 (6) the contribution from instantaneous diffusion would reduce to 9.6% of that caused by a π pulse when comparing to that raised by a π/5 pulse which will be considered as having diminished dipolar dephasing.

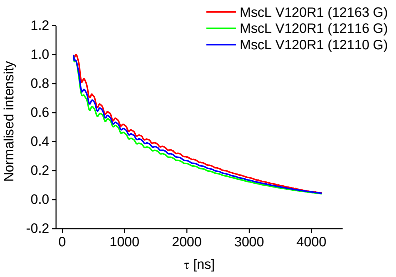

Figure S31: The magnetic field (B) was varied from 12090 to 12210 G, with 12116 G being the field where the signal maximum is found in the field swept EPR spectrum. As expected the higher the spectral intensity the higher the dephasing rate, with the fastest decay being measured at the field of maximum spectral intensity (12116 G).

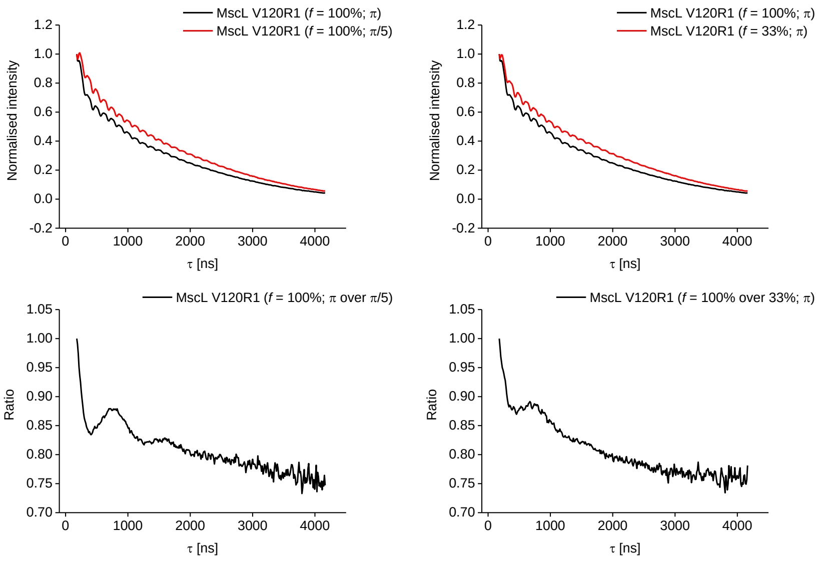

Figure S32: Decay traces from dipolar dephasing experiments on MscL V120R1 for the fully labeled sample (top left) and comparing π decay rates for the 100% vs. the 33% labeled sample (top right). Corresponding ratios are shown in the bottom row.

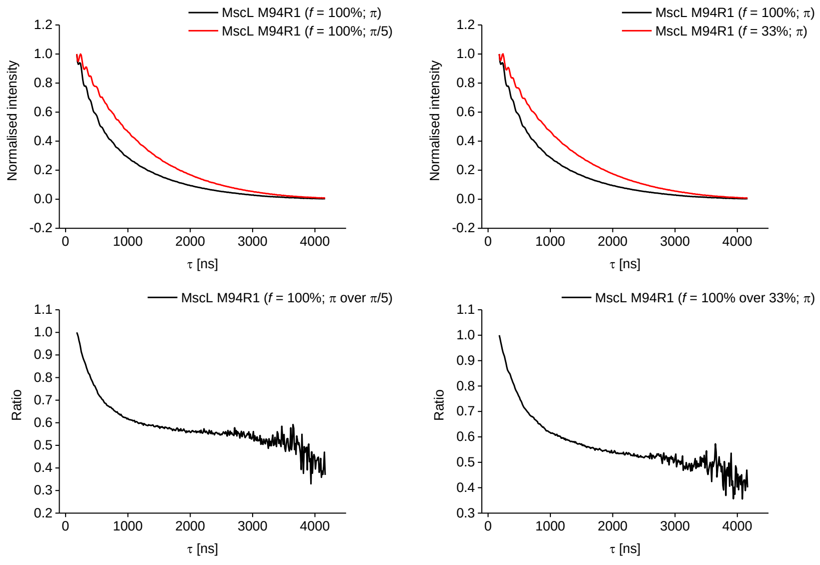

Figure S33: Decay traces from dipolar dephasing experiments on MscL M94R1 for the fully labeled sample (top left) and comparing π decay rates for the 100% vs. the 33% labeled sample (top right). Corresponding ratios are shown in the bottom row.

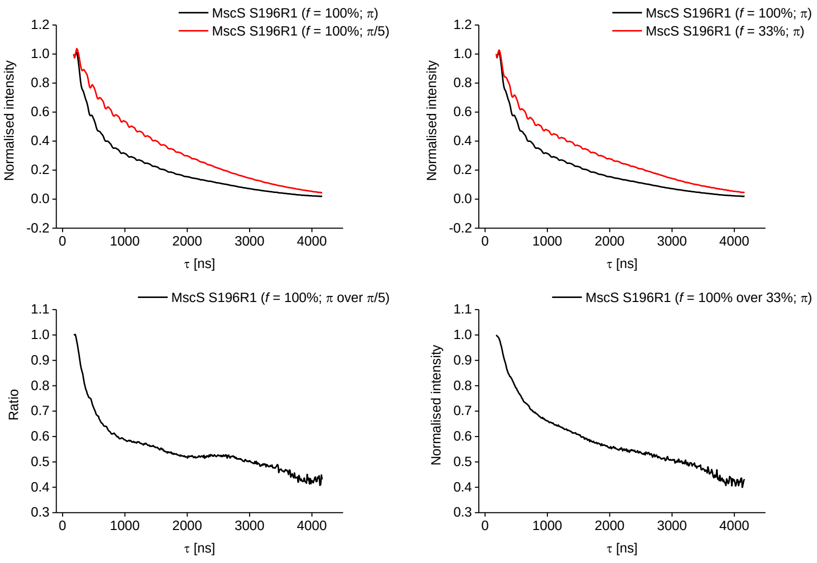

Figure S34: Decay traces from dipolar dephasing experiments on MscS S196R1 for the fully labeled sample (top left) and comparing π decay rates for the 100% vs. the 33% labeled sample (top right). Corresponding ratios are shown in the bottom row.

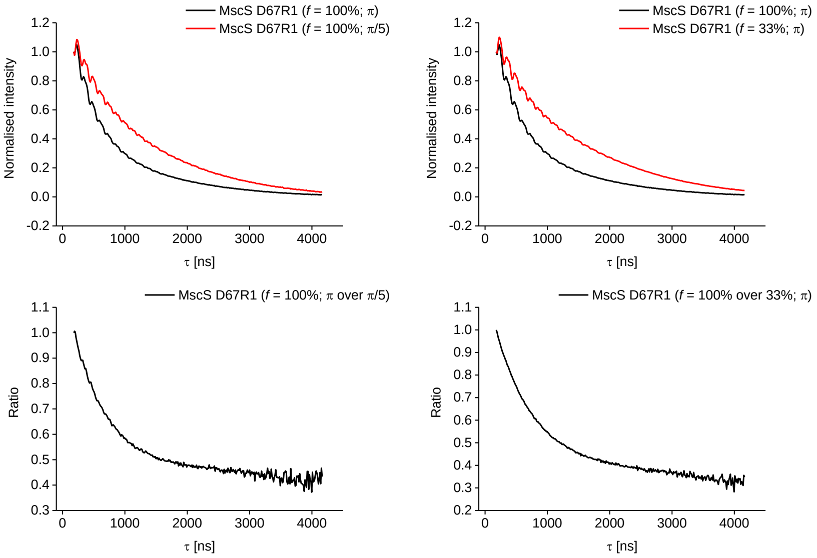

Figure S35: Decay traces from dipolar dephasing experiments on MscS D67R1 for the fully labeled sample (top left) and comparing π decay rates for the 100% vs. the 33% labeled sample (top right). Corresponding ratios are shown in the bottom row.

Chapter 4: Peak intensities

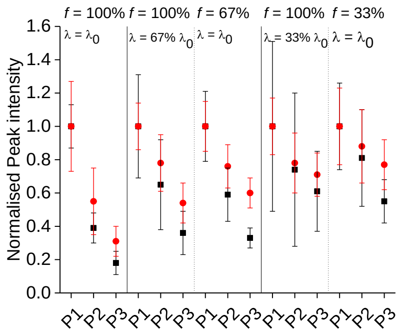

Figure S36: Peak integrals (mean ±2σ confidence intervals) for MscS S196R1. P1 = shortest, P2 = middle, P3 = longest distance peak; black: non-scaled data, red: power-scaled data.

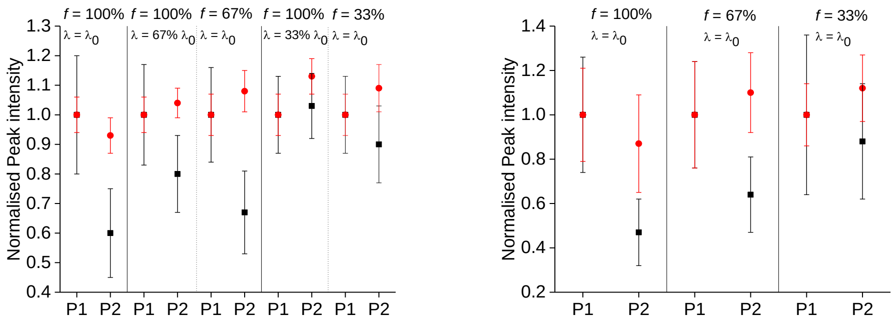

Figure S37: Peak integrals (mean ±2σ confidence intervals) for MscL V120R1. P1 = short, P2 = long distance peak. Left: Q-band; right: X-band; black: non-scaled data, red: power-scaled data.

Chapter 5: Supporting References

1. Krivov, G. G., M. V. Shapovalov, and R. L. Dunbrack, Jr. 2009. Improved prediction of protein side-chain conformations with SCWRL4. Proteins 77:778-795.
2. Polyhach, Y., E. Bordignon, and G. Jeschke. 2011. Rotamer libraries of spin labelled cysteines for protein studies. Phys. Chem. Chem. Phys. 13:2356-2366.
3. Hagelueken, G., R. Ward, J. H. Naismith , O. Schiemann. 2012. MtsslWizard: In Silico Spin-Labeling and Generation of Distance Distributions in PyMOL. Appl. Magn. Reson. 42:377-391.
4. Perozo, E., A. Kloda, D. M. Cortes , B. Martinac. 2001. Site-directed spin-labeling analysis of reconstituted Mscl in the closed state. J. Gen. Physiol. 118:193-206.
5. Jeschke, G., V. Chechik, P. Ionita, A. Godt, H. Zimmermann, J. Banham, C. R. Timmel, D. Hilger , H. Jung. 2006. DeerAnalysis2006 - a comprehensive software package for analyzing pulsed ELDOR data. Appl. Magn. Reson. 30:473-498.
6. Schweiger, A., and G. Jeschke. 2001. Principles of pulse electron paramagnetic resonance. OUP Oxford, New York.
