# Supplementary material for: Sparse Labeling PELDOR Spectroscopy on Multimeric Mechanosensitive Membrane Channels
Source: Biophys J. 2017 Nov 7;113(9):1968–78. doi: 10.1016/j.bpj.2017.09.005 (PMC5685675; doi:10.1016/j.bpj.2017.09.005)
Supplement: Document S2. Article plus Supporting Material [file mmc2.pdf]

Biophysical Journal
Article

Biophysical Society

Sparse Labeling PELDOR Spectroscopy on Multimeric Mechanosensitive Membrane Channels

Katrin Ackermann,[1,2] Christos Pliotas,[1,2] Silvia Valera,[1,2] James H. Naismith,[2] and Bela E. Bode[1,2,*]
[1]Centre of Magnetic Resonance and [2]Biomedical Sciences Research Complex and EaStCHEM School of Chemistry, University of St Andrews, North Haugh, St Andrews, Fife, United Kingdom

ABSTRACT Pulse electron paramagnetic resonance (EPR) is being applied to ever more complex biological systems comprising multiple subunits. Membrane channel proteins are of great interest as pulse EPR reports on functionally significant but distinct conformational states in a native environment without the need for crystallization. Pulse EPR, in the form of pulsed electron-electron double resonance (PELDOR), using site-directed spin labeling, is most commonly employed to accurately determine distances (in the nanometer range) between different regions of the structure. However, PELDOR data analysis is more challenging in systems containing more than two spins (e.g., homomultimers) due to distorting multispin effects. Without suppression of these effects, much of the information contained in PELDOR data cannot be reliably retrieved. Thus, it is of utmost importance for future PELDOR applications in structural biology to develop suitable approaches that can overcome the multispin problem. Here, two different approaches for suppressing multispin effects in PELDOR, sparse labeling of the protein (reducing the labeling efficiency f) and reducing the excitation probability of spins (λ), are compared on two distinct bacterial mechanosensitive channels. For both the pentameric channel of large conductance (MscL) and the heptameric channel of small conductance (MscS) of *Escherichia coli*, mutants containing a spin label in the cytosolic or the transmembrane region were tested. Data demonstrate that distance distributions can be significantly improved with either approach compared to the standard PELDOR measurement, and confirm that $\lambda < 1/(n-1)$ is needed to sufficiently suppress multispin effects (with n being the number of spins in the system). A clear advantage of the sparse labeling approach is demonstrated for the cytosolic mutants due to a significantly smaller loss in sensitivity. For the transmembrane mutants, this advantage is less pronounced but still useful for MscS, but performance is inferior for MscL possibly due to structural perturbations by the bulkier diamagnetic spin label analog.

INTRODUCTION

Pulse electron paramagnetic resonance (EPR) has become an important tool in structural biology. Most commonly, site-directed spin labeling (1,2) is used to introduce paramagnetic centers into a protein or nucleic acid, and the distance between these spin labels can be determined using a pulse EPR technique called “pulsed electron-electron double resonance” (PELDOR or “double electron-electron resonance”, DEER) (3–6)). PELDOR is highly complementary to other biophysical techniques employed in structural biology, most importantly offering the opportunity to accurately measure distances in the nanometer range (from 2 to 10 nm and beyond) (3,7). Compared to more established methods such as x-ray crystallography, Förster resonance energy transfer, or nuclear magnetic resonance spectroscopy, pulse EPR spectroscopy is not dependent on the growth of crystals, with measurements performed in frozen aqueous solutions; the PELDOR technique does not require the presence of different labels, and is not limited by rotational correlation times or the complexity of the system investigated.

Very briefly, in a PELDOR experiment the distance between paramagnetic centers in a given system is determined from the dipolar coupling between these centers (5,6). Thereby, the length of the dipolar evolution time t limits the maximum distance that can reliably be retrieved (4). The dipolar interaction, recorded as an oscillation during the dipolar evolution, is then processed to obtain the corresponding distance distribution. This is most usually done using a mathematical procedure called “Tikhonov regularization” within the DeerAnalysis software developed to

Submitted May 17, 2017, and accepted for publication September 5, 2017.
*Correspondence: beb2@st-andrews.ac.uk
Christos Pliotas’s present address is Biomedical Sciences Research Complex and School of Biology, University of St Andrews, North Haugh, St Andrews, Fife, United Kingdom.
Editor: David Cafiso.
https://doi.org/10.1016/j.bpj.2017.09.005
© 2017 Biophysical Society.
This is an open access article under the CC BY license (http://creativecommons.org/licenses/by/4.0/).

CrossMark

analyze PELDOR data (8). In this context, Tikhonov regularization is employed to best stabilize the solution of the moderately ill-posed inverse problem of going from time domain to distance data.

Over the past decade, the use of PELDOR applications has moved from small soluble proteins bearing two spins within the same polypeptide chain to ever more complex systems of considerable size and consisting of multiple subunits (3). Large membrane protein complexes are of interest for PELDOR measurements to assess conformational changes because obtaining a complete set of x-ray crystal and/or electron microscopy structures can be challenging (9–11). The approach of using an existing crystal structure in combination with pulse EPR measurements to assess functional changes has proven promising for membrane proteins (12–17). Pulse EPR has previously been employed to investigate the *Escherichia coli* small-conductance mechanosensitive channel MscS, a homoheptameric channel protein (18–20), including the role of the lipid environment on the gating of MscS (21). The immediate lipid environment has been shown to influence protein stability (22,23). The *E. coli* large-conductance mechanosensitive channel MscL, a homopentameric ion channel, for which no full-length crystal structure has been reported to date, has also been probed using continuous-wave EPR spectroscopy in both the open and the closed forms (24,25).

Homomultimeric proteins will most often be studied with a single spin-labeled cysteine residue per monomer. All oligomers higher than dimers will carry more than two spins leading to multispin effects (26) caused by sum and difference combinations of the dipolar frequencies involved (27). They often result in the suppression of the longer distances present within the distance distribution (28), and the appearance of ghost-peaks that make an unambiguous interpretation of the data more difficult (19,26,29); these effects worsen with increasing number of spins in the system (29). One approach to suppress multispin effects is power scaling, a postprocessing approach scaling the raw data (26). However, it has been demonstrated to not completely suppress multispin effects in systems with seven or more spins (19).

An experimental approach reducing the multispin effects by reducing the probability of exciting (i.e., pumping) spins (λ) (27) and thus, the probability of multiple simultaneous excitations, is called "λ-reduction". This, however, comes at the cost of overall sensitivity. Recently, we have demonstrated that λ-reduction, in combination with power scaling, can be used to successfully and sufficiently suppress multispin effects (19), in line with previous predictions (26), if λ is kept smaller than $1/(n-1)$, with n being the number of spins.

Finally, and unsurprisingly, multispin effects can be reduced by reducing the number of spins. This has been achieved in the past using an approach called "spin dilution", whereby spin-labeled protein was mixed with either wild-type (unlabeled) protein or protein with a diamagnetic spin label, forming heterodimers or higher oligomers via exchange or during reconstitution (28,30–34). However, this approach relies on the exchange of labeled and nonlabeled monomers, which is often not possible for proteins where no measurable exchange takes place (e.g., membrane proteins) and when the protein cannot be correctly refolded after denaturation into lower oligomers. In such cases, sparse labeling needs to be performed on the fully assembled protein complex, which can be achieved by either providing substoichiometric amounts of spin label for the number of sites present, or preferably a mixture of diamagnetic and paramagnetic spin label to achieve the desired labeling degree with respect to the paramagnetic label (labeling efficiency f), as large excesses of labeling reagent are often needed to react the large majority of sites (24,35). Recently, this approach has been successfully applied to a dimer of heptamers, i.e., a 14-spin system, where a combination of sparse labeling and λ-reduction was used to suppress multispin effects in PELDOR distance measurements (35). Alternative gadolinium-based spin labels were found to result in a lower susceptibility to multispin effects due to the lower achievable λ, and comparison with sparse labeling using conventional nitroxide labels (36) is very promising. These labels have also been employed in transmembrane peptides (37). The advent of Q-band and high field spectrometers and increased availability of the labels is likely to advance their mainstream application.

To date, no quantitative assessment of the separate influence of the two experimental approaches for suppression of multispin effects on overall measurement sensitivity and efficiency has been performed. Considering the uncertainties encountered for labeling transmembrane domains in membrane proteins, it is currently unclear how the sparse labeling approach would perform compared to λ-reduction.

The aim of this work is to apply the sparse labeling approach to both cytosolic and transmembrane mutants of MscL and MscS. The performance of sparse labeling and λ-reduction is compared by assessing sensitivities and the suppression of longer distances in the resulting distance distributions to obtain clear recommendations for PELDOR applications. Furthermore, results highlight structural information on the system that can be obtained from PELDOR experiments in addition to the mere distance distributions.

MATERIALS AND METHODS

Protein expression, purification, and spin labeling

The cytosolic (S196C) and transmembrane (D67C) single cysteine mutants of MscS were expressed, purified, and spin labeled as described previously (18). The cytosolic (V120C) and transmembrane (M94C) single cysteine mutants of MscL were obtained following a similar protocol, with the

only difference being that proteins were expressed in BL21(DE3) cells (Thermo Fisher Scientific, Paisley, UK), using a pET-52b vector. Protein concentrations were determined based on UV absorbance using NanoDrop (Thermo Fisher Scientific) as described previously (38); extinction coefficients ε were predicted using the ExPASy ProtParam tool (39) (calculations were based on the amino acid sequence of the protein (40,41), with improved accuracy to obtain an average deviation between observed and predicted ε of <3.2% for Trp-containing proteins and of 6.5% for Trp-less proteins (42)). Paramagnetic MTSL spin label and its NO-acetylated diamagnetic analog ((1-oxyl-2,2,5,5-tetramethyl-δ3-pyrroline-3-methyl) methanethiosulfonate (MTSL) and (1-acetoxy-2,2,5,5-tetramethyl-δ3-pyrroline-3-methyl) methanethiosulfonate (dMTSL); Toronto Research Chemicals, Ontario, Canada) were premixed for preparation of the sparsely labeled samples before performing the labeling. Achieved labeling degrees were highly reproducible and accurately determined using quantitative continuous-wave EPR or a thermal fluorescence emission assay as previously described (18,20,38).

EPR sample preparation, PELDOR measurements, and data analysis

Purified membrane proteins in dodecyl β-D maltopyranoside detergent micelles were measured in deuterated buffer with 50% deuterated ethylene glycol as cryoprotectant at a final protein (multimer) concentration of ~30–50 μM (monomer concentration ~150–350 μM). A quantity of 65 μL of the final sample mixture was transferred into 3-mm EPR quartz tubes (Wilmad-LabGlass, Vineland, NJ) and flash frozen in liquid nitrogen until use.

PELDOR measurements were recorded at X-band frequencies (~9.5 GHz, 1 kW) using a 3-mm split-ring (MS3) resonator or at Q-band frequencies (34 GHz, 150 W) using a 3-mm cylindrical resonator in TE012 mode (QT-II) as described previously (43). A frequency offset (pump – detection frequency) of −70 MHz at X-band and of +80 MHz at Q-band was used. Shot repetition times were set to between 2.5 and 3.0 ms; τ_1 was set to 380 ns, and τ_2 varied depending on the mutant and frequency. Pulse lengths used were 16 and 32 ns for $\pi/2$ and π detection, respectively, and 12 ns for the PELDOR π-pump pulse (for MscL M94 data recorded with the long time window shown in Supporting Material, the π-pump lengths varied from 12 to 20 ns). The pump pulse was placed on the maximum of the spectrum and on the resonance frequency of the resonator. Measurements with a reduced inversion efficiency (27), i.e., the probability of pumping spins (λ), were performed as described previously by keeping the length of the pump pulse constant while attenuating the pump power (19,27). The maximum achievable pump efficiency (λ_0) was estimated to be 0.25 at the Q-band setup used in this study, in good agreement with data on model systems (19).

PELDOR data were analyzed using the MATLAB (The MathWorks, Natick, MA) plugin DeerAnalysis2015 (8). Raw experimental data were background-corrected using a monoexponential decay function before subjecting the trace to Tikhonov regularization. The optimum regularization parameter α was chosen by visual inspection according to the L-curve criterion (44). For MscS, the excitation bandwidth filter was set to 16 MHz. For further statistical analysis of the data, the validation tool of DeerAnalysis2015 was employed. The background start time was varied from 5 to 80% of the dipolar evolution time, with trials every 5%, i.e., 16 trials; random noise was added at the level 1.50, i.e., adding 50% noise, with 50 trials, resulting in a total number of 800 trials per trace. Only data sets that were within 15% of the best (i.e., lowest) root mean square deviation (RMSD) observed were kept (default prune factor 1.15). In case the best RMSD corresponded to a fit with a rising background function (which would correspond to a negative sample concentration), this was attributed to an artifact at the end of the time trace and the experimental time window was cut by 10% and analysis was repeated. This cutting procedure was performed as often as required (maximum of three times corresponding to a 30% cut of the initial trace). Distance distributions are all plotted together with color bars indicating reliability ranges as derived from DeerAnalysis2015, with one exception. The software does not plot distances beyond the red reliability range, i.e., beyond $6 \times (t/2)^{1/3}$, whereas distance distributions shown in this study are all plotted between 1 and 8 nm for consistency.

Distance peak integrals were determined as follows. The upper and lower limits of the 2σ confidence intervals from statistical analysis as described above were integrated for each distance peak separately (integration boundaries: 1.44–3.02 nm, 3.02–4.44 nm, and 4.44–5.31 nm for MscS S196R1; 1.48–2.69 nm and 2.69–3.84 nm for MscL V120R1 (Q-band); and 1.47–2.67 nm and 2.67–3.87 nm for MscL V120R1 (X-band)). The area of the peak was taken as the mean of the two integrals, and the difference between the integrals is taken as the $\pm 2\sigma$ confidence interval, plotted as error bars in the Supporting Material. All values were normalized to the integral of the shortest distance. The errors given in Tables 1 and 2 are estimated by propagation of the uncertainties of the peak integrals.

Normalized sensitivity (S_N) values were calculated as the sensitivity (S) divided by the number of echoes per point, with the latter being calculated as the product of number of scans, shots per point, number of tau averages, and phase cycle. S is determined as the ratio of modulation depth Δ over the absolute experimental noise when the trace is normalized to one. Here, Δ is given in DeerAnalysis2015 (Δ_{exp}). Determining the noise level of the fit RMSD can be misled by poor fits. Instead, the noise level was taken from the imaginary part of raw PELDOR traces after phase correction.

Δ_{fit} was obtained from fitting λ_0 globally for all experiments on a given labeled protein at a given frequency per Eq. 1 (27,29,45), with f and λ set to $f_0/3$, $2f_0/3$, or f_0 and $\lambda_0/3$, $2\lambda_0/3$, or λ_0, respectively, in dependence on experiment and sample. All fitted modulation depths were obtained assuming quantitative labeling ($f_0 = 1$) in good agreement with biochemical data (20,38). The value Δ_{fit} is then calculated using the best fit values of λ_0:

$$\Delta = 1 - (1 - \lambda f)^{n-1}. \quad (1)$$

In cases where complexes with different numbers of electron spins are contributing differently to the signal (46–48), a summation over all contributions with a weighting factor $x(k)$ can be performed:

$$\Delta = 1 - \frac{\sum_{k=1}^{n} \binom{n}{k} f^k (1-f)^{n-k} (1-\lambda)^{k-1} k\, x(k)}{\sum_{k=1}^{n} \binom{n}{k} f^k (1-f)^{n-k} k\, x(k)}. \quad (2)$$

Here, the contributions for the individual k-fold labeled n-mers are calculated by the product of the respective binomial coefficient, the probability of k labels on n labeling sites, the modulation depth of a k-fold labeled species, the number of labels (as each can be the detected one), and the relative weight $x(k)$ of the k-fold labeled species. The residual offsets (1-Δ) for all k-fold labeled n-mers are added and normalized by their contribution to the signal at zero time. For the case of all x being equal, Eq. 2 reverts to Eq. 1.

Dipolar contributions to echo modulation and dephasing were qualitatively assessed by measuring Hahn echo decays varying the flip angle of the second pulse from $\beta = \pi$ to $\beta = \pi/5$ for fully labeled samples. The influence of the spectral intensity on the signal decay (using $\beta = \pi$) was investigated exemplary for MscL V120R1 by varying the magnetic field incrementally from 12,090 G to 12,210 G, with 12,116 G being the maximum of the field swept EPR spectrum.

Modeling

Distance distributions were predicted using the MATLAB plugin MMM2015.1 in combination with the third-party software SCWRL4 (49,50) and the PyMOL plugin MtsslWizard (51). For MscS, PDB: 5AJI

was used (spin-labeled mutant D67R1 with resolved lipid acyl chains) (21). The R1 residue (corresponding to a cysteine spin labeled with MTSL) was converted to cysteine in MMM before the site-scan at position 67, and distance distributions were predicted in presence and absence of lipids. MtsslWizard settings were "painstaking and tight" for MscS D67R1 and "painstaking and loose" for MscS S196 (because no conformers were found for the "tight" setting), for both mutants the distance distributions were predicted in the presence and absence of lipids for comparison. The crystal structure of the spin-labeled mutant D67R1 of MscS (PDB: 5AJI) was used to obtain direct distance measurements from the position of the radical (approximated to be localized on the oxygen atom of the nitroxyl group), allowing for a direct comparison with modeling results. PDB: 4LKU (52) was used for predicting distance distributions for the cytosolic part of the *E. coli* MscL (i.e., V120R1). No *E. coli* crystal structure of the transmembrane portion of the channel has been reported to date. Therefore, the x-ray crystal structure from the *Mycobacterium tuberculosis* MscL ortholog (PDB: 2OAR (53)) was used for modeling the distances of the transmembrane residue. MTSL was modeled onto residue F88, which by sequence alignment corresponds to *E. coli* M94 (25). For both MscL mutants MtsslWizard settings were "painstaking and tight". All modeling in MMM2015.1 was performed at ambient temperature settings (298 K), and obtained distance distributions were compared with and without repacking of the side chains (the SCWRL function (49) to correct side-chain conformations in the crystal structure that could be different in solution).

RESULTS AND DISCUSSION

Modeling of distance distributions

In general, distance distributions predicted by MMM and MtsslWizard were in good agreement with each other. The good agreement between PELDOR results and direct distance measurements between the radical pairs of MTSL in the crystal structure of the spin-labeled mutant MscS D67R1 (21) has been noted. In MscS, the presence of lipids made no difference to the calculated distance distributions. The structure used for modeling distance distributions in MscL did not contain any resolved or modeled lipids. For both proteins, repacking of the side chains in MMM did not significantly change the predicted distance distribution. Detailed modeling results can be found in the Supporting Material. In the following, predicted distance distributions from MMM (repacked, no lipids) and MtsslWizard (no lipids) are shown together with experimental results (*solid* and *dashed gray lines*, respectively).

PELDOR on sparsely labeled cytosolic mutants of multisubunit membrane proteins

PELDOR experiments were performed for a cytosolic mutant of MscS, with the MTSL label attached to residue 196 (S196R1), corresponding to seven spins per protein complex under fully labeled conditions (Fig. 1, *top left*).

The resulting multispin effects (26) have previously led to focusing on the first (shortest) interspin distance while omitting more ambiguous data for longer distances (18,20). Experimental PELDOR data confirm that even with power scaling (26), only the first (shortest) distance can be reliably retrieved from fully labeled protein (Fig. 1, *top right*). Additional approaches to tackle the multispin problem are λ-reduction (19,26), or reducing the percentage of spin label per protein complex (35). Although efficiency of the latter has not been systematically evaluated, we have recently estimated keeping λ below $1/(n-1)$ in combination with power scaling as the uppermost limit to sufficiently suppress multispin effects in a given n-spin system (19), in agreement with earlier predictions (4,26). For the heptameric MscS, this means $\lambda < 1/6$, corresponding to $\lambda < 67\%\ \lambda_0$ under our conditions at Q-band (estimating a λ_0 of 0.25). Thus, fully labeled MscS S196R1 was investigated at $\lambda = 67\%\ \lambda_0$ and in addition at $\lambda = 33\%\ \lambda_0$ (corresponding to $\lambda = 0.08$) (Fig. 1, *middle*) to investigate if results could be improved by further reduction of λ. These results were compared against the alternative approach using sparsely labeled protein at labeling ratios (% of paramagnetic label) of 2/3 ($f = 67\%$) and 1/3 ($f = 33\%$) (Fig. 1, *bottom*).

Data demonstrate that both sparse labeling and λ-reduction are effective at suppressing multispin effects observed in the heptameric protein MscS. Using either method, both the second and the third distances can be reliably retrieved. At $f = 67\%$ and $\lambda = 67\%\ \lambda_0$ power scaling is required, whereas at $f = 33\%$ and $\lambda = 33\%\ \lambda_0$, even without

TABLE 1 Assessment of Sparse Labeling versus λ-Reduction for MscS S196R1

% Label	% λ_0	Δ_{exp}	Δ_{fit}	S_N	DD %
100	100	0.630	0.687	1.46	55 ± 12
100	33	0.224	0.304	0.64	78 ± 11
33	100	0.462	0.304	1.37	88 ± 15
100	67	0.431	0.527	1.12	78 ± 10
67	100	0.629	0.527	1.79	76 ± 8

Given are calculated and experimental modulation depths Δ_{fit} and Δ_{exp} and normalized sensitivity values (S_N). For the corresponding power-scaled distance distributions (*DD*), the percentage of the second distance integral with respect to the first distance integral is given (± error) as an indication for the recovery of intensities of longer distance peaks. The maximum λ achieved experimentally at full labeling was ~0.15 (expected ~0.25, see main text); λ_0 obtained via global fitting (Eq. 1) was found to be 0.176.

TABLE 2 Assessment of Sparse Labeling versus λ-Reduction for MscL V120R1

X/Q	% Label	% λ_0	Δ_{exp}	Δ_{fit}	S_N	DD %
Q	100	100	0.695	0.690	10.05	93 ± 4
Q	100	33	0.204	0.298	3.73	113 ± 5
Q	33	100	0.377	0.298	5.98	109 ± 5
Q	100	67	0.443	0.523	7.83	104 ± 4
Q	67	100	0.607	0.523	8.30	108 ± 5
X	100	100	0.817	0.882	0.40	87 ± 14
X	67	100	0.712	0.725	0.26	110 ± 16
X	33	100	0.523	0.447	0.26	112 ± 11

See Table 1 for details. The maximum λ achieved experimentally at full labeling was ~0.26 at Q-band (expected ~0.25) and ~0.35 at X-band (expected ~0.4); λ_0 obtained via global fitting (Eq. 1) was found to be 0.254 (Q-band) and 0.413 (X-band).

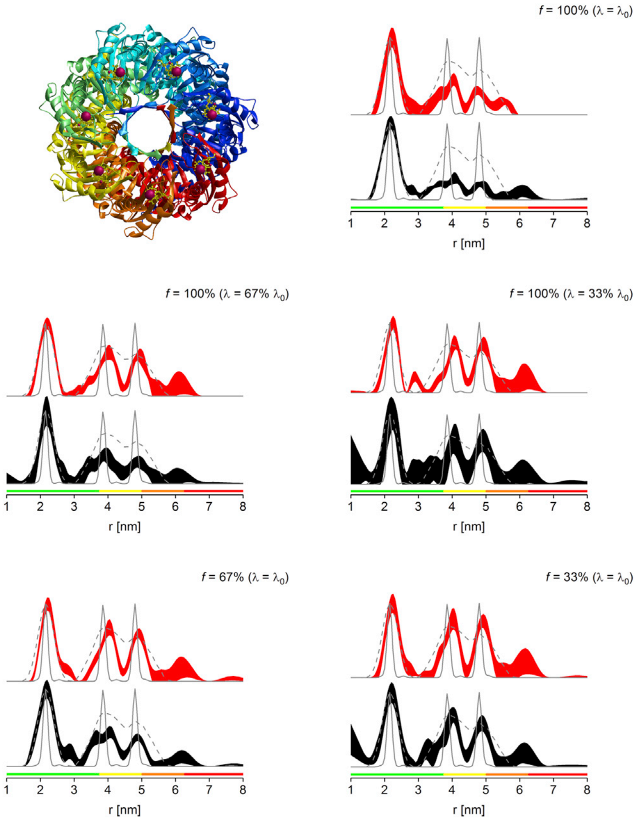

FIGURE 1 MscS S196R1 PELDOR distance distributions obtained for fully labeled protein (*top right*), λ-reduced fully labeled protein (*middle panel*, *left*, $\lambda = 67\%\ \lambda_0$; *right*, $\lambda = 33\%\ \lambda_0$), and sparsely labeled protein (*bottom panel*, *left*, $f = 67\%$; *right*, $f = 33\%$). Depicted are the 2σ confidence intervals as black and red areas for nonscaled and power-scaled data, respectively. Predicted distance distributions from MMM2015.1 and MtsslWizard are shown as gray lines (*solid* and *dashed*, respectively). The bottom color bars represent reliability ranges for the distance distributions (8) (*green*, shape reliable; *yellow*, mean and width reliable; *orange*, mean reliable; *red*, no quantification possible). See Supporting Material for raw PELDOR data and processing. (*Top left*) Shown here is the MMM model of fully labeled protein. To see this figure in color, go online.

power scaling, the three distances can be retrieved as predicted previously (19), although with larger uncertainties.

These results agree with the previous finding that keeping $\lambda < 1/(n-1)$ (19) is a prerequisite for efficient suppression of multispin effects in fully labeled systems. The slight but visible improvement of the data at even lower f or λ indicate that the finding made for fully labeled systems can be extended so that $\lambda f = 1/(n-1)$ should indeed be regarded as the uppermost limit. Comparing λ-reduction and sparse labeling results by eye suggests that the latter may be slightly more efficient, as evidenced by smaller uncertainties (narrower confidence intervals) after statistical analysis. Multispin effects lead to a loss of intensity in long-range distances (29). Vice versa, the efficient suppression of multispin effects can be demonstrated by recovery of the appropriate distance peak intensities for all peaks. Thus, these intensities were determined by integration (Table 1 and Supporting Material), instead of comparing peak maxima, which can be misleading due to different peak widths. Because the corresponding errors are estimated from validations of distance distributions with substantial noise added, they are likely to be overestimated. Nevertheless, a recovery of peak intensities can be observed with both λ-reduction and sparse labeling, and within errors no significant differences between the two approaches can be found. However, normalized sensitivity values (S_N) clearly show a substantial advantage of sparse labeling over λ-reduction (Table 1), with only small to moderate losses compared to the fully labeled protein, and approximately twice the sensitivity at $f = 33\%$ compared to $\lambda = 33\%\ \lambda_0$.

It should be noted that the λ achieved for MscS S196R1 at full labeling is considerably lower than the expected one of 0.25; instead, the value obtained is ~0.15. However, this is consistent with previous observations on MscS (18–20). Data further demonstrate that the error in the accuracy for setting the pump efficiency λ was <10%, in line with previous data (~10–15%) (27). The achieved maximum λ for fully labeled MscS S196R1 was highly reproducible both within and in-between samples (0.15–0.16 and 0.14, respectively), and the achieved reduced $\lambda = 33\%\ \lambda_0$ was highly consistent (i.e., 0.04–0.05). The possible causes for the anomalies in the observed modulation depths will be discussed later.

The second cytosolic mutant investigated is V120R1 of MscL, a homopentameric protein. Thus, a λ of $1/(5-1) = 0.25$ (19) would be expected to be the upper limit for suppression of multispin effects if used in combination with power scaling. This limiting λ of 0.25 is close to the value found for λ_0 for biradicals with the Q-band setup available in St Andrews (150 W amplifier and 3 mm sample access). This means measuring a pentameric protein such as MscL under these conditions (note that higher λ_0 may be observed in other setups (54)) using the maximum achievable λ, and analyzing the data after power scaling, abolishes the need for either λ-reduction or sparse labeling although still efficiently suppressing multispin effects. Our data confirm this prediction, demonstrating that the expected peak integrals (and thus, longer-range distances) can already be retrieved at $f = 100\%$ and λ_0 if power-scaled (Fig. 2, and Supporting Material).

The discrepancies between the models and the experimental distance distributions are attributed to the fact that the models are based on the structure of a truncated protein, and might suggest less steric bulk than present in the full-length construct.

Notably, as observed for MscS S196R1, the loss in normalized sensitivity S_N is much greater for λ-reduction than for sparse labeling (Table 2).

For MscL V120R1 we demonstrate that no λ-reduction or sparse labeling is required at Q-band to suppress multispin effects; however, for this protein, these detrimental effects can be observed at a higher λ_0, as present at X-band (Fig. 3 and

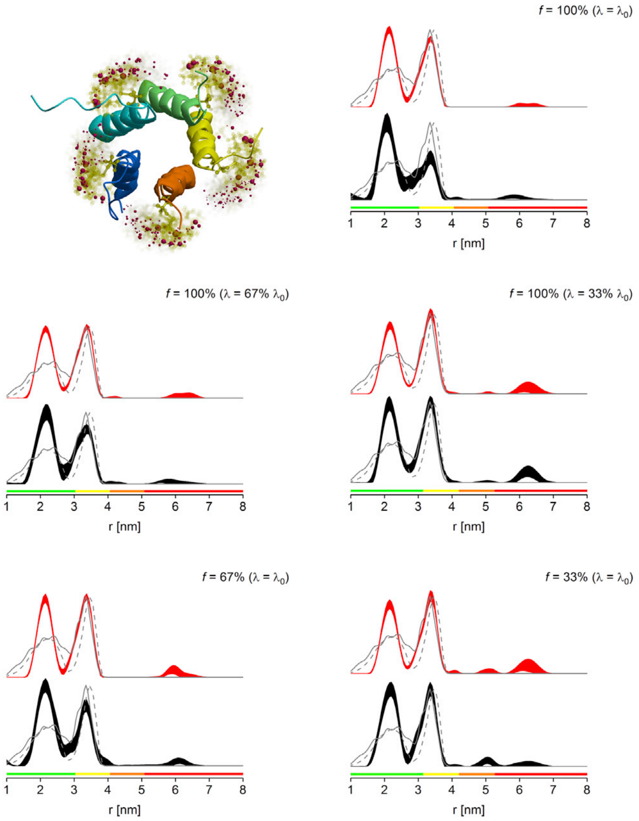

FIGURE 2 MscL V120R1 PELDOR distance distributions obtained for fully labeled protein at Q-band (*top right*), λ-reduced fully labeled protein (*middle panel*, *left*, $\lambda = 67\%\ \lambda_0$; *right*, $\lambda = 33\%\ \lambda_0$), and sparsely labeled protein (*bottom panel*, *left*, $f = 67\%$; *right*, $f = 33\%$). See Fig. 1 for details on the 2σ confidence intervals, predicted distance distributions, and color bars. See Supporting Material for raw PELDOR data and processing. (*Top left*) Shown here is the MMM model of fully labeled cytosolic domain of *E. coli* MscL protein. To see this figure in color, go online.

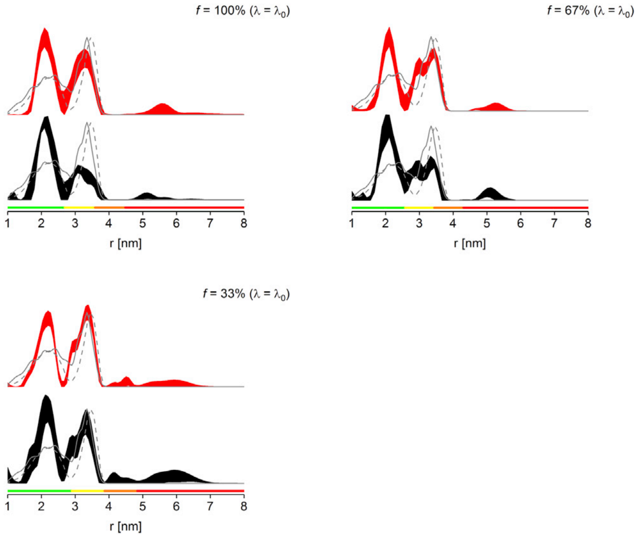

FIGURE 3 MscL V120R1 PELDOR distance distributions obtained for fully labeled protein at X-band (*top left*), and sparsely labeled protein (*top right*, $f = 67\%$; *bottom left*, $f = 33\%$). See Fig. 1 for details on the 2σ confidence intervals, predicted distance distributions, and color bars. See Supporting Material for raw PELDOR data and processing. To see this figure in color, go online.

Supporting Material). The slightly distorted distance distributions can be rescued using sparse labeling in combination with power scaling, thereby allowing us to fully retrieve the intensity of the second distance peak, as evidenced by comparison of the peak integrals. Interestingly, the power scaling of data taken at reduced λ or f seems to overestimate the intensity of this peak (Table 2 and Supporting Material).

Data shown here for both cytosolic mutants confirm our previous findings regarding the maximum λ to suppress multispin effects (19). If feasible, λ should be reduced even further, which can be achieved using λ-reduction and/or sparse labeling. Although both methods have been used in a combined fashion recently (35), to our knowledge, this is the first study to directly compare efficiencies and sensitivities of these two methods. No significant differences were observed in terms of peak integrals (as a proxy for the quality of the distance distributions); however, sparse labeling exhibited a much higher sensitivity than corresponding measurements using λ-reduction, rendering it the preferred option for both systems investigated here. Furthermore, data obtained from statistical analyses (i.e., 95% confidence bands) demonstrate that it is advisable to always use the appropriate power scaling (26) when investigating multispin systems because, at least for all cases tested here with f or λ reduced, this consistently reduces the uncertainty and improves recovery of longer distances. Interestingly, power scaling slightly increased the confidence intervals for the fully labeled MscS S196R1 measured at 100% λ_0. However, for this case, power scaling alone does not fully recover all peak intensities, so that reduction of f or λ has to be strongly recommended, confirming the conclusion that power scaling reduces the uncertainty in all relevant cases.

PELDOR on sparsely labeled transmembrane mutants of multisubunit membrane proteins

Whereas cytosolic mutants can be expected to have, in general, a high labeling efficiency due to accessibility of the label to the cysteine site, this is not the case for transmembrane mutants, where the presence of lipids or detergents may inhibit labeling. To the best of our knowledge, no report so far has investigated sparse labeling for PELDOR at transmembrane sites of membrane proteins. Here, the heptameric MscS D67R1 mutant was used to compare sparse labeling and λ-reduction approaches for the suppression of multispin effects.

The distance distributions obtained from PELDOR experiments on the MscS D67R1 transmembrane mutant (Fig. 4 and Supporting Material) demonstrate the improvement that can be achieved using either approach compared to analyzing the fully labeled protein at full λ. Both approaches allow recovering the second and—to a certain extent—the third

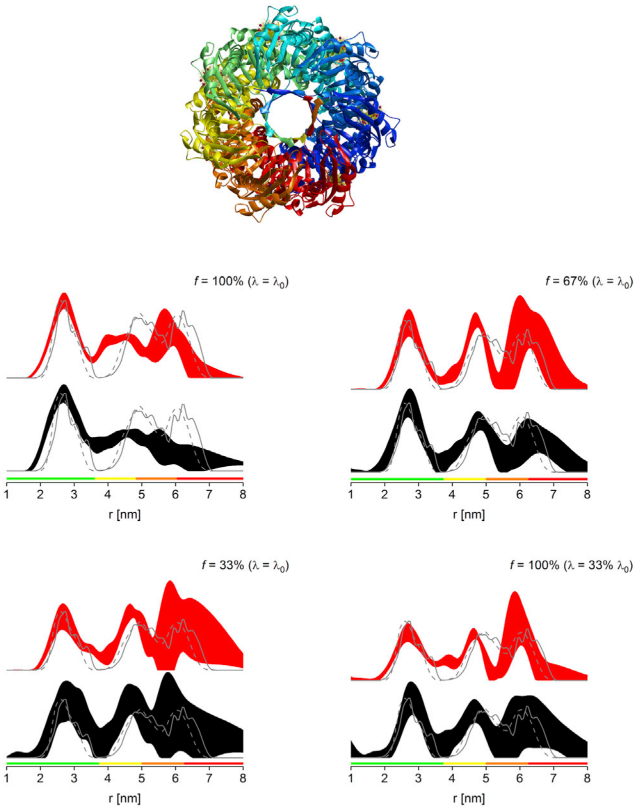

FIGURE 4 MscS D67R1 PELDOR distance distributions obtained for fully labeled protein (*middle left*), sparsely labeled protein (*bottom left*, $f = 33\%$; *middle right*, $f = 67\%$), and λ-reduced fully labeled protein (*bottom right*, $\lambda = 33\%\ \lambda_0$). See Fig. 1 for details on the 2σ confidence intervals, predicted distance distributions, and color bars. See Supporting Material for raw PELDOR data and processing. (*Top*) Shown here is the MMM model of fully labeled protein. To see this figure in color, go online.

distance. Compared to both cytosolic mutants, the maximum (third) distance for MscS D67R1 is longer and thus, would require an extended dipolar evolution time to be resolved reliably. However, with the time achieved here, the longest distance is located partly within the red reliability range, indicating that quantification is no longer possible. Therefore, we refrained from determining peak integrals for this mutant.

The sensitivity comparison (Table 3) suggests that the two approaches yield similar results, but it should be noted that the λ estimated was slightly lower than expected (0.13 vs. 0.15 in MscS S196R1), indicating that the labeling efficiency f_0 could be slightly lower compared to the cytosolic mutant. The modulation depths will be discussed in the next section. By far the best-resolved distance distribution was obtained using the 67% labeled mutant, which also showed approximately double the sensitivity of the fully labeled version and a higher signal-to-noise of the trace (Supporting Material). Reducing labeling efficiency beyond the required maximum leads to a loss in both signal magnitude and modulation depth. Both will reduce linearly in the regime of low labeling efficiencies. Thus, the sensitivity is expected to scale with the square of the labeling degree. Surprisingly, this scaling is already observed when reducing f from 67 to 33% in MscS D67R1, but this might be overestimated by the fact only the 67% sample filled the entire active volume of the resonator, whereas the other two samples were underfilled by 20–25%. Further uncertainties arise from the achievable f_0 in the transmembrane region.

Taken together, data demonstrate that even for a transmembrane mutant, sparse labeling was at least as good as λ-reduction for suppression of multispin effects.

A second transmembrane site for investigation was chosen from the pentameric system MscL. Here, no *E. coli* crystal structure is available for the transmembrane region. To obtain an approximate distance distribution for the chosen mutant M94R1, the MTSL label was modeled onto the corresponding residue (F88) from *M. tuberculosis*, as determined from sequence alignment (25).

Similarly to MscS D67R1, the expected long distance peak for MscL M94R1 lies partly in the red reliability range (Fig. 5 and Supporting Material). The power-scaled distance distribution obtained for the 33% labeled protein seems to indicate the presence of a distance peak in that region, but better data with longer dipolar evolution times would be required to draw any firm conclusions on this. Due to the higher uncertainties for the longer distance arising from the limited dipolar evolution time, achievable peak integrals were not determined in analogy to MscS D67R1.

For the first distance peak, a distribution substantially broader than predicted is consistently observed. Apart from some discrepancies arising from the different organism used for modeling, there are two possible explanations for this finding. One is the simultaneous presence of different oligomeric states of the protein (pentamer and tetramer) (24,55–57). Although under our experimental conditions the data for the cytosolic mutant of MscL, V120R1, corresponded precisely to the results modeled for a pentameric structure, it cannot be excluded that another mutant (such as M94R1) would exhibit a different oligomeric state as a direct effect of the mutation itself. However, we have no further indication for such a case.

The second possibility is the simultaneous presence of different structural states of the protein, e.g., the presence of

TABLE 3 Assessment of Sparse Labeling versus λ-Reduction for MscS D67R1

% Label	% λ_0	Δ_{exp}	Δ_{fit}	S_N
100	100	0.558	0.601	0.62
67	100	0.496	0.449	1.17
100	33	0.234	0.252	0.32
33	100	0.274	0.252	0.33

Given are calculated and experimental modulation depths Δ_{fit} and Δ_{exp} and normalized sensitivity values (S_N). The maximum λ achieved experimentally at full labeling was ~0.13 (expected ~0.25, see main text); λ_0 obtained via global fitting (Eq. 1) was found to be 0.142.

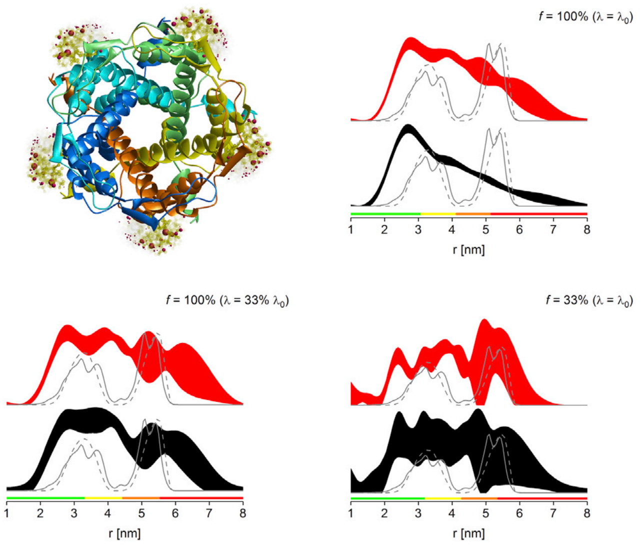

FIGURE 5 MscL M94R1 PELDOR distance distributions obtained for fully labeled protein (*top right*), λ-reduced fully labeled protein (*bottom left*, $\lambda = 33\%\ \lambda_0$), and sparsely labeled protein (*bottom right*, $f = 33\%$). See Fig. 1 for details on the 2σ confidence intervals, predicted distance distributions, and color bars. See Supporting Material for raw PELDOR data and processing. (*Top left*) Shown here is the MMM model of fully labeled protein using MscL from *M. tuberculosis* (label modeled onto residue F88). To see this figure in color, go online.

the closed state of the channel together with additional conducting states, as suggested previously (24,58). No conclusions on this can be drawn from data for the cytosolic mutant, V120R1, because this region of the protein is expected to be structurally unaffected by the presence of different functional states. However, such a mixture of different states would affect the observed distance distribution for the transmembrane region, and could therefore well account for the observed very broad distribution for the first distance peak resembling two (or more) distinct conformational states. Thus, MscL M94R1 data shown here could indicate the presence of multiple functional states, in line with previous observations (24,58). However, one has to bear in mind that the absence of lateral tension of the lipid bilayer (i.e., in dodecyl β-D maltopyranoside) may result in a higher conformational flexibility of the protein. Thus, the protein may sample a larger range of distances than the ones expected from simple analysis of the *M. tuberculosis* structure, which might also explain the observed broader distribution. Therefore, further studies are needed to clarify this issue.

Interestingly, sensitivity of the 33% labeled sample was only about half of the sensitivity observed for the corresponding λ-reduced measurement of the fully labeled sample (Table 4), which contrasts with our observations for all other mutants analyzed, both cytosolic and transmembrane. One possible explanation for this unexpectedly low sensitivity could be the interference of the label with close-by lipids. It has been observed that MTSL at position M94

TABLE 4 Assessment of Sparse Labeling versus λ-Reduction for MscL M94R1

% Label	% λ_0	Δ_{exp}	Δ_{fit}	S_N
100	100	0.711	0.638	3.20
100	33	0.228	0.267	0.98
33	100	0.178	0.267	0.48

For details, see Table 3. The maximum λ achieved experimentally at full labeling was ~0.27 (expected ~0.25); λ_0 obtained via global fitting (Eq. 1) was found to be 0.224.

was rigid rather than mobile, with the involvement of immobilizing lipids as a potential reason (25).

In our hands, from all mutants and proteins tested in this study, MscL M94C not only produced the lowest expression yield, but was also the only mutant where dMTSL, in contrast to MTSL, caused protein destabilizing effects, potentially leading to a degree of dissociation. A lower than estimated multimer concentration will result in an overall reduced sensitivity due to a reduction in signal. This destabilizing effect could be due to the residue either being directly involved in lipid binding or being close to a lipid binding site as suggested (25), and the bulkier dMTSL might negatively affect lipid binding and thus, protein stability (23,59). Importantly, this would have no impact on the fully labeled sample, but only on the sparsely labeled sample, as observed experimentally when comparing sensitivities for the λ-reduced fully labeled with the sparsely labeled sample.

The direct effect of both sparse labeling and λ-reduction on the recorded PELDOR data is illustrated in the scaled fits of the power-scaled traces (Supporting Material), indicating the presence of multispin effects due to the changes of the trace curvatures with different degrees of f and λ, as demonstrated previously (29).

Comparing modulation depths and sensitivities for λ-reduction versus sparse-labeling

Reducing either labeling or λ reveals already from the primary data that the experimental modulation depth does indeed decrease. Interestingly, whereas the normalized sensitivity S_N always decreased upon reducing the pumping efficiency λ, in two cases an increase was observed upon reducing f (both MscS mutants with $f = 67\%$; see Tables 1 and 3). Phase shift and echo reduction are known to decrease with a weaker pump pulse and this will aid the signal strength. The latter will also reduce less than the nominal spin concentration when reducing f as dipolar dephasing reduces (see instantaneous diffusion/dipolar dephasing below). However, the reduced modulation depth Δ will counteract these effects with respect to S_N. In all experiments performed on MscS, the observed modulation depths were systematically smaller than expected from theory and previous experimental λ-values (60,61). Thus, in an internally consistent approach we approximate all λ_0 to be identical for one mutant. Fitting λ_0 for all experiments on

one mutant and frequency band allows then recalculating an expected Δ_{fit}. Two trends prevail—the Δ_{fit} for all sparse labeling experiments at Q-band (apart from MscL M94R1) are overestimating the experimental values, whereas underestimating all reduced λ experiments. Indeed, the described differences in reduced λ versus reduced f are significant beyond experimental uncertainties, with the modulation depths for the sparsely labeled samples at $f = 33\%$ being about a factor 2 larger compared to the corresponding λ-reduced samples for both cytosolic mutants (Tables 1 and 2). Exemplary error estimates for MscS S196R1 obtained by propagating errors in λ and f (10 and 5%, respectively—see Materials and Methods) yield relative uncertainties of 7, 27 or 16% in Δ (for $f = 1$ and $\lambda = \lambda_0$, $f = 1$ and $\lambda = 33\%\ \lambda_0$, or $f = 33\%$ and $\lambda = \lambda_0$) and thus cannot explain observed differences or anomalies of a factor of 2 in experimentally obtained modulation depths.

From Eq. 2 follows that reduced λ_0 should display a larger experimental modulation depth than reducing labeling by the same degree if the signal contributions are increasingly reduced with a rising number of spin labels per complex. However, the observed trend in the experiment is opposite. There are several possible reasons for this: 1) The value λ could be overestimated and thus reduced further than intended; this is unlikely, as our model system work shows reasonable agreement with expectations (19). 2) Sparse labeling could be underestimated by a preferential labeling with MTSL over dMTSL; however, this would need to be systematic for all mutants of both proteins. 3) The modulation depth is estimated in the limit that all dipolar modulations decay to zero. In contrast, the difference between very similar frequencies will be close to zero frequency and will not interfere to zero but to a constant offset. This effect could be exacerbated by orientation selection leading to the excitation of a smaller range of dipolar couplings. It seems unlikely to have similarly pronounced orientation selection in both systems. 4) Dipolar echo modulation or dephasing will reduce the contribution of complexes with many spins (46–48). This arises from the loss of echo signal by unwanted flips of close-by electron spins during the π-pulses of the detection sequence leading to incomplete refocusing. In a PELDOR experiment this will reduce the refocused echo signal and this will be multiplicative for additional B-spins (46). This leads to an increased contribution of complexes with fewer spin labels in sparse labeling experiments that should decrease the modulation depth further rather than increase it. This could be modeled by Eq. 2, but it is contradicting the experimental trend so this model is not feasible here. If, however, there are some contributions from free spin labels or nonspecifically bound labels, these will dephase more slowly than fully labeled complexes and reduce the overall modulation depth. Here, these dipolar dephasing effects were measured to estimate the influence of the proximity of multiple spins on sensitivity. Results suggest that the signal decay has an oscillatory and a diffusive component owed to intra- and intermolecular dipolar couplings (see Supporting Material for details). Traces where the dipolar contributions to transverse dephasing were reduced to $<10\%$ closely resemble those of the 33% sparsely labeled samples. At experimentally relevant settings (pulse separation of 4 μs corresponding to a typical dipolar evolution time used here) the dipolar dephasing contribution reduces the signal of the fully labeled samples of MscS and MscL M94R1 by $>50\%$, whereas MscL V120R1 is merely reduced by 25%. This can only in part be attributed to intramolecular dephasing and will be further enhanced by the reduction in spin-label concentration (in this study, when sparse labeling, the protein concentration is kept constant so that the spin label concentration is proportional to the labeling degree). Thus, sparsely labeled samples do not suffer from dephasing as much as the corresponding fully labeled samples that can almost fully compensate the loss in signal as demonstrated for MscS S196R1. It is important to stress that the reduction in concentration does not manifest to be as detrimental as one might have suspected initially. To first approximation, both the signal and Δ will reduce linearly with f. On the other hand, the signal will be enhanced by a reduced instantaneous diffusion rate. This S_N gain can even overcompensate the loss in S_N that is inverse square with f depending on the pulse sequence length (determining the dipolar evolution time accessible) and the local spin concentration. Reducing f will reduce both the background decay rate and instantaneous diffusion losses. In contrast, reducing λ will only reduce the background decay rate. This constitutes a clear difference between the two approaches and outlines why sparse labeling will be particularly advantageous at high concentrations or extended dipolar evolution times. This is in good agreement with results on the cytosolic mutants, although transmembrane mutants are expected to bear higher uncertainty in labeling efficiencies.

Taken together, data show that sparse labeling results in larger modulation depths and sensitivity than λ-reduction. Dipolar dephasing studies reveal that the sensitivity in fully labeled samples is compromised by the high local spin concentration. However, the effect of dipolar dephasing on the modulation depths in sparsely labeled samples contradicts predictions and remains the subject of future studies.

CONCLUSIONS

In this work, two approaches for suppressing multispin effects in oligomeric proteins, λ-reduction and sparse labeling, were assessed and quantified on a set of multimeric membrane proteins using both cytosolic and transmembrane mutants. The results demonstrate substantial improvement of the distance distributions achievable using either

approach when compared to no such steps. Our data suggest sparse labeling is the preferred method, with higher sensitivity retained compared to the corresponding λ-reduced experiment, at least for cytosolic mutants, where labeling efficiency is more easily controllable than in transmembrane mutants. The potential interference with lipid binding of the MscL M94 mutant suggests that, apart from the actual labeling efficiency, the labeling site also needs to be chosen carefully for transmembrane mutants. As the diamagnetic label analog is bulkier than MTSL, even greater care has to be taken to avoid structural perturbations at crowded labeling sites, such as lipid binding pockets.

The clear advantages of suppressing multispin effects are demonstrated by retrieving not only the first but also the second and (partly) even the third distance peak in MscS. The V120 mutant of MscL exemplifies the role of the achievable λ for multispin effects by illustrating the differences observed between the excitation bandwidths at X- and Q-band. For all proteins investigated here, our previously suggested upper limit for λ of $1/(n-1)$ (19), in combination with power scaling holds, and can be expanded to $\lambda f < 1/(n-1)$. Data presented here suggest that sparse labeling to achieve the required λf is preferable to a fully labeled protein measured using a reduced λ; however, in cases where a very large number of spins is present, it might be necessary to combine both approaches to obtain a sufficiently reduced λ (35).

Pulse EPR spectroscopy and specifically PELDOR distance measurements have become a very important tool in structural biology, and pushing the limits of this method will further expand the scope of applications.

The research data supporting this publication can be accessed at https://doi.org/10.17630/0cc71494-7e2f-49f3-bc98-c3b3768a4c65.

SUPPORTING MATERIAL

Supporting Materials and Methods and thirty-seven figures are available at http://www.biophysj.org/biophysj/supplemental/S0006-3495(17)31014-7.

AUTHOR CONTRIBUTIONS

B.E.B., J.H.N., and C.P. designed the research. K.A., C.P., S.V., J.H.N., and B.E.B. performed research and analyzed data. K.A. and B.E.B. wrote the manuscript with contributions from all authors.

ACKNOWLEDGMENTS

The authors acknowledge Ms. Elyse Fischer for help during sample preparation, and Dr. Akiko Rasmussen and Prof. Ian R Booth for the provision of initial reagents.

B.E.B. is grateful for funding from the European Union (Marie Curie Actions REA 334496). This work was supported by the Engineering and Physical Sciences Research Council (EPSRC) (EP/M024660/1) and the Wellcome Trust (099149/Z/12/Z). C.P. is a Royal Society of Edinburgh (RSE) Personal Research Fellow, funded by the Scottish Government.

SUPPORTING CITATIONS

Reference (62) appears in the Supporting Material.

REFERENCES

1. Altenbach, C., S. L. Flitsch, …, W. L. Hubbell. 1989. Structural studies on transmembrane proteins. 2. Spin labeling of bacteriorhodopsin mutants at unique cysteines. *Biochemistry.* 28:7806–7812.
2. Hubbell, W. L., C. J. López, …, Z. Yang. 2013. Technological advances in site-directed spin labeling of proteins. *Curr. Opin. Struct. Biol.* 23:725–733.
3. Jeschke, G. 2012. DEER distance measurements on proteins. *Annu. Rev. Phys. Chem.* 63:419–446.
4. Jeschke, G. 2014. Interpretation of dipolar EPR data in terms of protein structure. *Struct. Bonding.* 152:83–120.
5. Milov, A. D., K. M. Salikov, and M. D. Shirov. 1981. Application of Endor in electron-spin echo for paramagnetic center space distribution in solids. *Fiz. Tverd. Tela.* 23:975–982.
6. Pannier, M., S. Veit, …, H. W. Spiess. 2000. Dead-time free measurement of dipole-dipole interactions between electron spins. *J. Magn. Reson.* 142:331–340.
7. Bowman, A., C. M. Hammond, …, T. Owen-Hughes. 2014. The histone chaperones Vps75 and Nap1 form ring-like, tetrameric structures in solution. *Nucleic Acids Res.* 42:6038–6051.
8. Jeschke, G., V. Chechik, …, H. Jung. 2006. DeerAnalysis2006—a comprehensive software package for analyzing pulsed ELDOR data. *Appl. Magn. Reson.* 30:473–498.
9. Bleicken, S., G. Jeschke, …, E. Bordignon. 2014. Structural model of active Bax at the membrane. *Mol. Cell.* 56:496–505.
10. Georgieva, E. R., P. P. Borbat, …, O. Boudker. 2013. Conformational ensemble of the sodium-coupled aspartate transporter. *Nat. Struct. Mol. Biol.* 20:215–221.
11. Joseph, B., A. Sikora, …, T. F. Prisner. 2015. Distance measurement on an endogenous membrane transporter in *E. coli* cells and native membranes using EPR spectroscopy. *Angew. Chem. Int.Engl.* 54:6196–6199.
12. Altenbach, C., A. K. Kusnetzow, …, W. L. Hubbell. 2008. High-resolution distance mapping in rhodopsin reveals the pattern of helix movement due to activation. *Proc. Natl. Acad. Sci. USA.* 105:7439–7444.
13. Dawidowski, D., and D. S. Cafiso. 2013. Allosteric control of syntaxin 1a by Munc18-1: characterization of the open and closed conformations of syntaxin. *Biophys. J.* 104:1585–1594.
14. Hilger, D., H. Jung, …, G. Jeschke. 2005. Assessing oligomerization of membrane proteins by four-pulse DEER: pH-dependent dimerization of NhaA Na^+/H^+ antiporter of *E. coli. Biophys. J.* 89:1328–1338.
15. Jeschke, G., C. Wegener, …, H. J. Steinhoff. 2004. Interresidual distance determination by four-pulse double electron-electron resonance in an integral membrane protein: the Na^+/proline transporter PutP of *Escherichia coli. Biophys. J.* 86:2551–2557.
16. Kazmier, K., S. Sharma, …, H. S. Mchaourab. 2014. Conformational cycle and ion-coupling mechanism of the Na^+/hydantoin transporter Mhp1. *Proc. Natl. Acad. Sci. USA.* 111:14752–14757.
17. Puljung, M. C., H. A. DeBerg, …, S. Stoll. 2014. Double electron-electron resonance reveals cAMP-induced conformational change in HCN channels. *Proc. Natl. Acad. Sci. USA.* 111:9816–9821.
18. Pliotas, C., R. Ward, …, J. H. Naismith. 2012. Conformational state of the MscS mechanosensitive channel in solution revealed by pulsed electron-electron double resonance (PELDOR) spectroscopy. *Proc. Natl. Acad. Sci. USA.* 109:E2675–E2682.
19. Valera, S., K. Ackermann, …, B. E. Bode. 2016. Accurate extraction of nanometer distances in multimers by pulse EPR. *Chemistry.* 22:4700–4703.

20. Ward, R., C. Pliotas, …, O. Schiemann. 2014. Probing the structure of the mechanosensitive channel of small conductance in lipid bilayers with pulsed electron-electron double resonance. *Biophys. J.* 106:834–842.
21. Pliotas, C., A. C. Dahl, …, J. H. Naismith. 2015. The role of lipids in mechanosensation. *Nat. Struct. Mol. Biol.* 22:991–998.
22. Battle, A. R., P. Ridone, …, B. Martinac. 2015. Lipid-protein interactions: lessons learned from stress. *Biochim. Biophys. Acta.* 1848:1744–1756.
23. Gupta, K., J. A. C. Donlan, …, C. V. Robinson. 2017. The role of interfacial lipids in stabilizing membrane protein oligomers. *Nature.* 541:421–424.
24. Perozo, E., D. M. Cortes, …, B. Martinac. 2002. Open channel structure of MscL and the gating mechanism of mechanosensitive channels. *Nature.* 418:942–948.
25. Perozo, E., A. Kloda, …, B. Martinac. 2001. Site-directed spin-labeling analysis of reconstituted MscL in the closed state. *J. Gen. Physiol.* 118:193–206.
26. von Hagens, T., Y. Polyhach, …, G. Jeschke. 2013. Suppression of ghost distances in multiple-spin double electron-electron resonance. *Phys. Chem. Chem. Phys.* 15:5854–5866.
27. Jeschke, G., M. Sajid, …, A. Godt. 2009. Three-spin correlations in double electron-electron resonance. *Phys. Chem. Chem. Phys.* 11:6580–6591.
28. Junk, M. J., H. W. Spiess, and D. Hinderberger. 2011. DEER in biological multispin-systems: a case study on the fatty acid binding to human serum albumin. *J. Magn. Reson.* 210:210–217.
29. Giannoulis, A., R. Ward, …, B. E. Bode. 2013. PELDOR in rotationally symmetric homo-oligomers. *Mol. Phys.* 111:2845–2854.
30. Aitha, M., T. K. Richmond, …, M. W. Crowder. 2014. Dilution of dipolar interactions in a spin-labeled, multimeric metalloenzyme for DEER studies. *J. Inorg. Biochem.* 136:40–46.
31. Langen, R., J. M. Isas, …, W. L. Hubbell. 1998. Membrane-mediated assembly of annexins studied by site-directed spin labeling. *J. Biol. Chem.* 273:22453–22457.
32. Rodriguez, F., S. L. Rouse, …, J. R. Schnell. 2013. Structural model for the protein-translocating element of the twin-arginine transport system. *Proc. Natl. Acad. Sci. USA.* 110:E1092–E1101.
33. Stone, K. M., J. Voska, …, S. Han. 2013. Structural insight into proteorhodopsin oligomers. *Biophys. J.* 104:472–481.
34. Xu, Q., J. F. Ellena, …, D. S. Cafiso. 2006. Substrate-dependent unfolding of the energy coupling motif of a membrane transport protein determined by double electron-electron resonance. *Biochemistry.* 45:10847–10854.
35. Schmidt, T., M. A. Wälti, …, G. M. Clore. 2016. Long distance measurements up to 160 Å in the GroEL tetradecamer using Q-band DEER EPR spectroscopy. *Angew. Chem. Int.Engl.* 55:15905–15909.
36. Edwards, D. T., T. Huber, …, S. Han. 2014. Determining the oligomeric structure of proteorhodopsin by Gd^{3+}-based pulsed dipolar spectroscopy of multiple distances. *Structure.* 22:1677–1686.
37. Matalon, E., T. Huber, …, D. Goldfarb. 2013. Gadolinium(III) spin labels for high-sensitivity distance measurements in transmembrane helices. *Angew. Chem. Int.Engl.* 52:11831–11834.
38. Branigan, E., C. Pliotas, …, J. H. Naismith. 2013. Quantification of free cysteines in membrane and soluble proteins using a fluorescent dye and thermal unfolding. *Nat. Protoc.* 8:2090–2097.
39. ProtParam. Swiss Institute of Bioinformatics, Lausanne, Switzerland. http://web.expasy.org/tools/protparam/.
40. Gill, S. C., and P. H. von Hippel. 1989. Calculation of protein extinction coefficients from amino acid sequence data. *Anal. Biochem.* 182:319–326.
41. Edelhoch, H. 1967. Spectroscopic determination of tryptophan and tyrosine in proteins. *Biochem.* 6:1948–1954.
42. Pace, C. N., F. Vajdos, …, T. Gray. 1995. How to measure and predict the molar absorption coefficient of a protein. *Protein Sci.* 4:2411–2423.
43. Kerry, P. S., H. L. Turkington, …, B. E. Bode. 2014. Analysis of influenza A virus NS1 dimer interfaces in solution by pulse EPR distance measurements. *J. Phys. Chem. B.* 118:10882–10888.
44. Chiang, Y. W., P. P. Borbat, and J. H. Freed. 2005. The determination of pair distance distributions by pulsed ESR using Tikhonov regularization. *J. Magn. Reson.* 172:279–295.
45. Bode, B. E., R. Dastvan, and T. F. Prisner. 2011. Pulsed electron-electron double resonance (PELDOR) distance measurements in detergent micelles. *J. Magn. Reson.* 211:11–17.
46. Jeschke, G. 2016. Dipolar spectroscopy—double-resonance methods. *eMagRes.* 5:1459–1476.
47. Salikhov, K. M., and I. T. Khairuzhdinov. 2015. Four-pulse ELDOR theory of the spin A1/2 label pairs extended to overlapping EPR spectra and to overlapping pump and observer excitation bands. *Appl. Magn. Reson.* 46:67–83.
48. Salikhov, K. M., I. T. Khairuzhdinov, and R. B. Zaripov. 2014. Three-pulse ELDOR theory revisited. *Appl. Magn. Reson.* 45:573–619.
49. Krivov, G. G., M. V. Shapovalov, and R. L. Dunbrack, Jr. 2009. Improved prediction of protein side-chain conformations with SCWRL4. *Proteins.* 77:778–795.
50. Polyhach, Y., E. Bordignon, and G. Jeschke. 2011. Rotamer libraries of spin labelled cysteines for protein studies. *Phys. Chem. Chem. Phys.* 13:2356–2366.
51. Hagelueken, G., R. Ward, …, O. Schiemann. 2012. MtsslWizard: in silico spin-labeling and generation of distance distributions in PyMOL. *Appl. Magn. Reson.* 42:377–391.
52. Walton, T. A., and D. C. Rees. 2013. Structure and stability of the C-terminal helical bundle of the *E. coli* mechanosensitive channel of large conductance. *Protein Sci.* 22:1592–1601.
53. Steinbacher, S., R. Bass, …, D. C. Rees. 2007. Structures of the prokaryotic mechanosensitive channels MscL and MscS. *Curr. Top. Membr.* 58:1–24.
54. Polyhach, Y., E. Bordignon, …, G. Jeschke. 2012. High sensitivity and versatility of the DEER experiment on nitroxide radical pairs at Q-band frequencies. *Phys. Chem. Chem. Phys.* 14:10762–10773.
55. Chang, G., R. H. Spencer, …, D. C. Rees. 1998. Structure of the MscL homolog from *Mycobacterium tuberculosis*: a gated mechanosensitive ion channel. *Science.* 282:2220–2226.
56. Liu, Z., C. S. Gandhi, and D. C. Rees. 2009. Structure of a tetrameric MscL in an expanded intermediate state. *Nature.* 461:120–124.
57. Reading, E., T. A. Walton, …, C. V. Robinson. 2015. The effect of detergent, temperature, and lipid on the oligomeric state of MscL constructs: insights from mass spectrometry. *Chem. Biol.* 22:593–603.
58. Sukharev, S. I., W. J. Sigurdson, …, F. Sachs. 1999. Energetic and spatial parameters for gating of the bacterial large conductance mechanosensitive channel, MscL. *J. Gen. Physiol.* 113:525–540.
59. Iscla, I., G. Levin, …, P. Blount. 2004. Defining the physical gate of a mechanosensitive channel, MscL, by engineering metal-binding sites. *Biophys. J.* 87:3172–3180.
60. Bode, B. E., D. Margraf, …, O. Schiemann. 2007. Counting the monomers in nanometer-sized oligomers by pulsed electron-electron double resonance. *J. Am. Chem. Soc.* 129:6736–6745.
61. Milov, A. D., A. B. Ponomarev, and Y. D. Tsvetkov. 1984. Electron-electron double resonance in electron spin echo: model biradical systems and the sensitized photolysis of decalin. *Chem. Phys. Lett.* 110:67–72.
62. Schweiger, A., and G. Jeschke. 2001. Principles of Pulse Electron Paramagnetic Resonance. Oxford University Press, New York, NY.

Biophysical Journal, Volume *113*

Supplemental Information

Sparse Labeling PELDOR Spectroscopy on Multimeric Mechanosensitive Membrane Channels

Katrin Ackermann, Christos Pliotas, Silvia Valera, James H. Naismith, and Bela E. Bode

Table of Contents

Chapter 1: Modeling results page 3
Chapter 2: Additional PELDOR data page 7
- 2.1) MscS S196R1 (cytosolic mutant)
- 2.2) MscL V120R1 (cytosolic mutant)
- 2.3) MscS D67R1 (transmembrane mutant)
- 2.4) MscL M94R1 (transmembrane mutant)

Chapter 3: Dipolar dephasing page 21
Chapter 4: Peak intensities page 24
Chapter 5: Supporting references page 25

Chapter 1: Modeling results

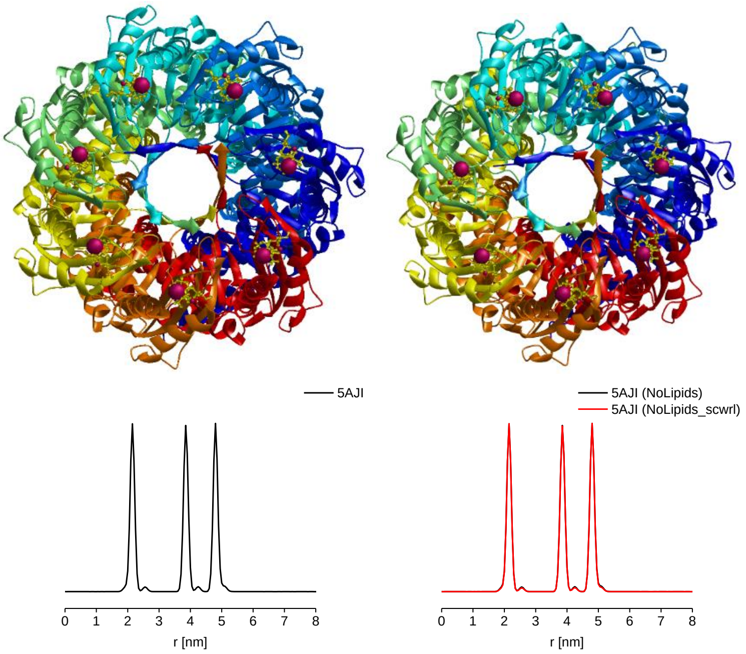

Figure S1: Modeling results for MscS S196R1 based on crystal structure pdb 5aji generated using MMM2015 (1,2). Top: Models with spin-label attached (left: pdb 5aji; right: pdb 5aji with lipids removed and side-chains repacked). Monomers are individually colored. Bottom: Distance distributions comparing the original pdb with the lipids removed and the side-chains repacked (scwrl). Side-chain repacking was not possible with the lipids still present. Virtually no differences between models and distance distributions can be observed.

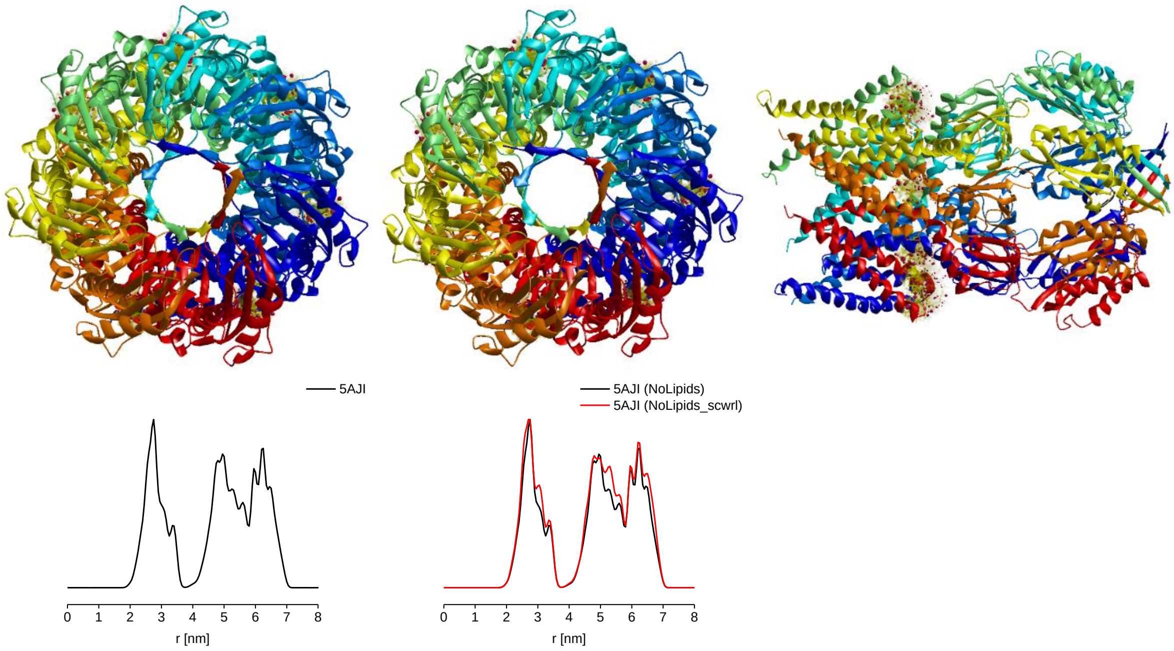

Figure S2: Modeling results for MscS D67R1 based on crystal structure pdb 5aji generated using MMM2015 (1,2). The spin-labelled residue at position 67 was mutated to a cysteine before the site-scan. Top: Models with spin-label attached (left: pdb 5aji; middle (top view) and right (side view): pdb 5aji with lipids removed and side-chains repacked). Monomers are individually colored. Bottom: Distance distributions comparing the original pdb with the lipids removed and the side-chains repacked (scwrl). Side-chain repacking was not possible with the lipids still present. Only minor differences between the distance distributions can be observed.

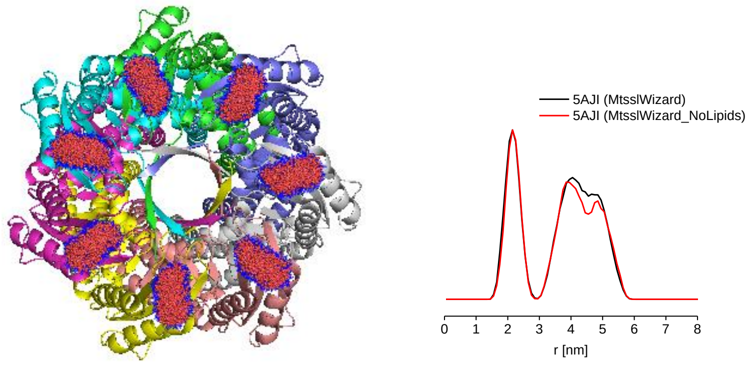

Figure S3: Modeling results for MscS S196R1 based on crystal structure pdb 5aji generated using MtsslWizard (3). Left: model with spin-labels attached; monomers are individually colored. Right: Distance distributions with and without lipids obtained for painstaking search at “loose” van-der-Waals cut-off settings (2.5 angstroms cut-off and 5 clashes allowed) as with the “tight” setting (3.4 angstroms cut-off and no clashes allowed) no rotamers were found. This “loose” setting might be attributable to the broader distribution compared to MMM.

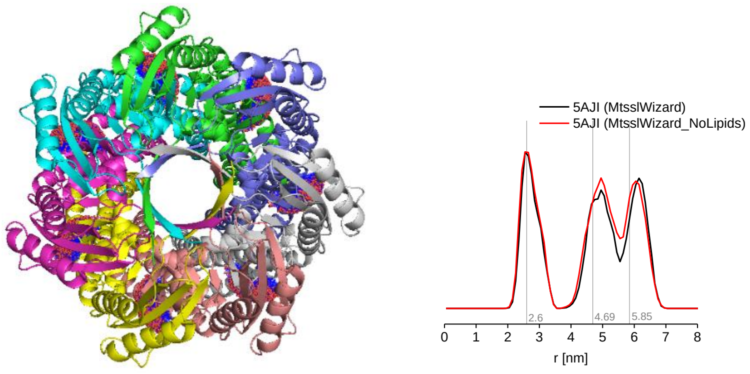

Figure S4: Modeling results for MscS D67R1 based on crystal structure pdb 5aji generated using MtsslWizard (3). Left: model with spin-labels attached; monomers are individually colored. Right: Distance distributions with and without lipids obtained for painstaking search at “tight” van-der-Waals cut-off settings. Only minor differences were observed. Vertical lines indicate the direct distance measurement from the position of the radical (approximated to be localized on the oxygen atom of the nitroxyl group) in the crystal structure in PyMOL; exact measurements are given in nm.

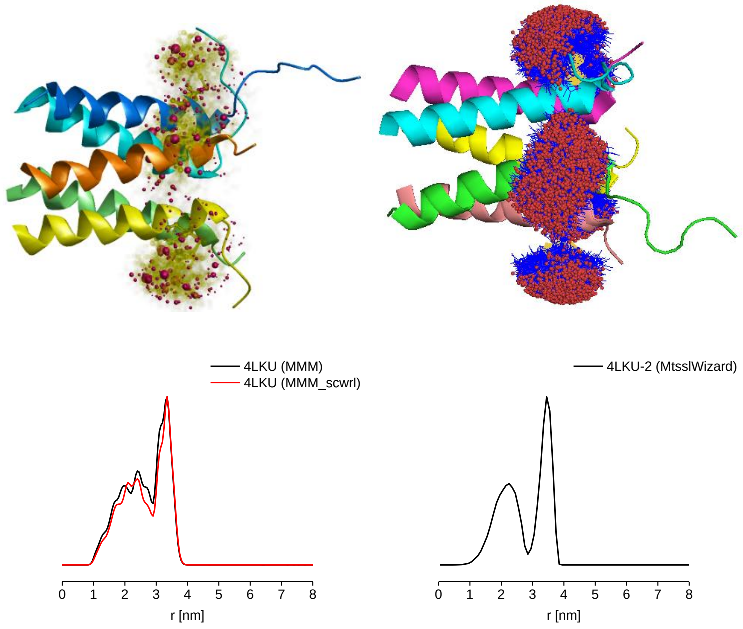

Figure S5: Top: Models of the cytosolic domain of *E.coli* MscL V120R1 based on crystal structure pdb 4lku generated using MMM2015.1 (left, side-chains re-packed) or MtsslWizard (right). Monomers are individually colored. Bottom: Corresponding distance distributions, in the case of MMM (left) with (scwrl) and without re-packing of side-chains. Only minor differences can be observed.

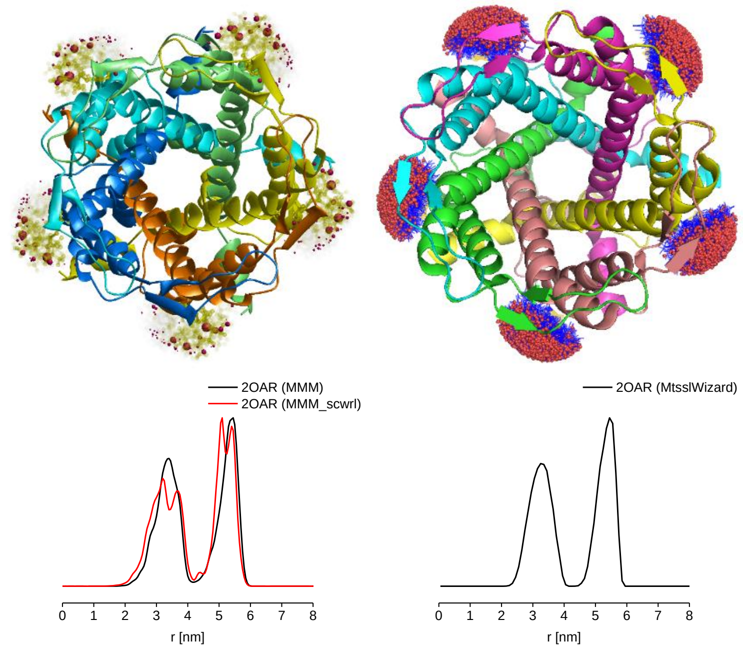

Figure S6: Top: Models of *M. tuberculosis* MscL F88R1 (corresponding to residue M94 in *E. coli* (4)) based on crystal structure pdb 2oar generated using MMM2015.1 (left, side-chains re-packed) or MtsslWizard (right). Monomers are individually colored. Bottom: Corresponding distance distributions, in the case of MMM (left) with (scwrl) and without re-packing of side-chains. Only minor differences can be observed.

Chapter 2: Additional PELDOR data

With the exception of Figure S30, all figures in this chapter are arranged in a similar way. Raw PELDOR data are shown on the top left. Background-corrected data with fit are shown on the top right. Corresponding distance distributions can be found on the bottom left, whereby the gray vertical line indicates the cut-off distance (at r /nm = (t /µs × 52)$^{1/3}$). The 95% confidence interval (±2σ) of the distance distributions obtained by statistical analysis is shown on the bottom right overlaid with the structural model in light gray. Fits and distance distributions are shown both, without (black) and with (red) power-scaling. All distance distributions are shown with color bars representing respective reliability ranges as described in detail in the DeerAnalysis manual (5) (green: shape reliable; yellow: mean and width reliable; orange: mean reliable; red: no quantification possible).

2.1) MscS S196R1 (cytosolic mutant)

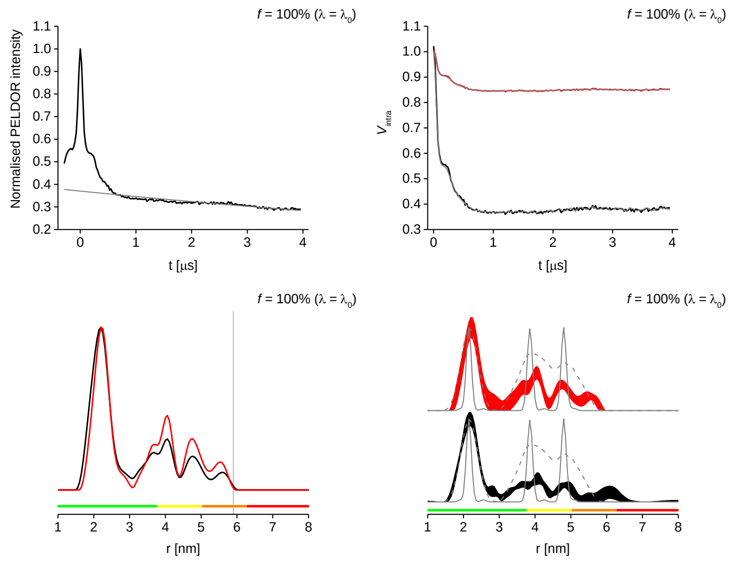

Figure S7: Q-band PELDOR data for MscS S196R1 at 100% labeling, full λ. See page 6 for details.

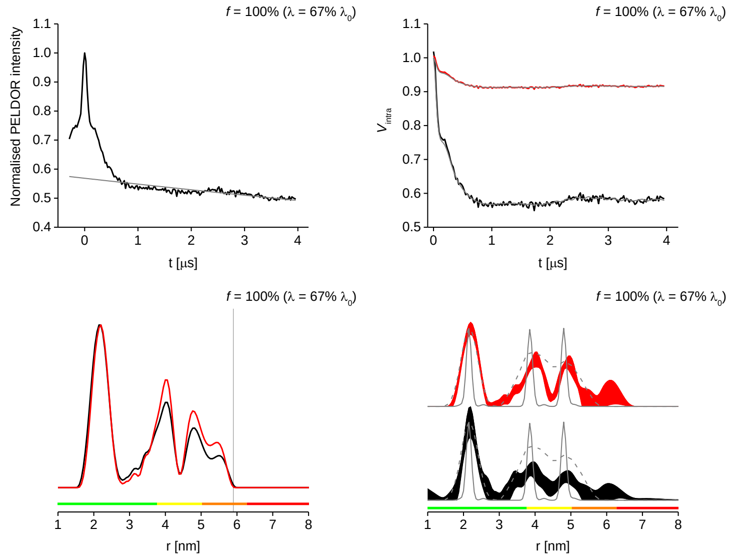

Figure S8: Q-band PELDOR data for MscS S196R1 at 100% labeling, $\lambda = 67\% \lambda_0$. See page 6 for details.

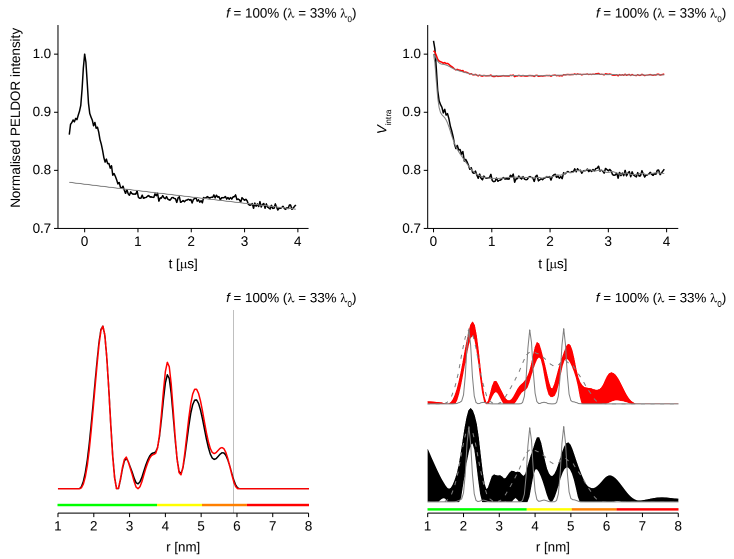

Figure S9: Q-band PELDOR data for MscS S196R1 at 100% labeling, $\lambda = 33\% \lambda_0$. See page 6 for details.

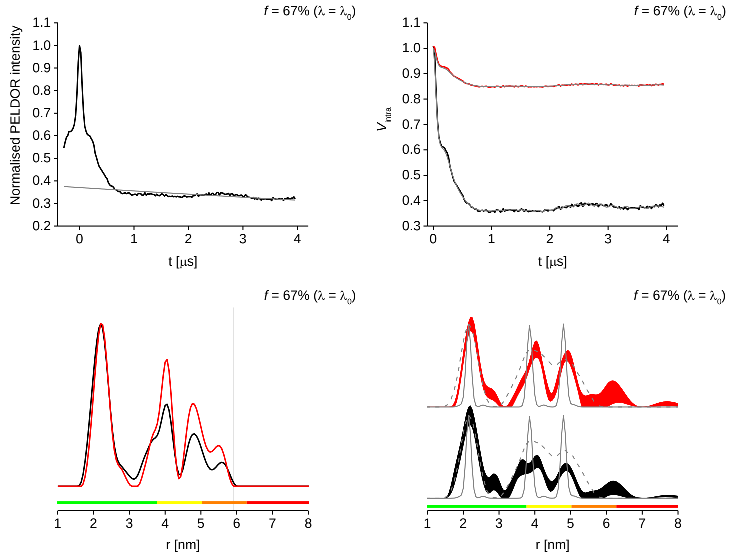

Figure S10: Q-band PELDOR data for MscS S196R1 at 67% labeling, full λ. See page 6 for details.

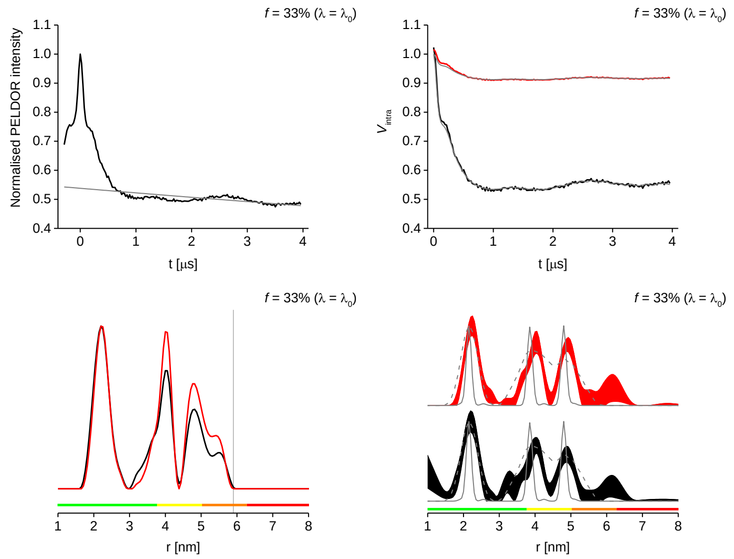

Figure S11: Q-band PELDOR data for MscS S196R1 at 33% labeling, full λ. See page 6 for details.

2.2) MscL V120R1 (cytosolic mutant)

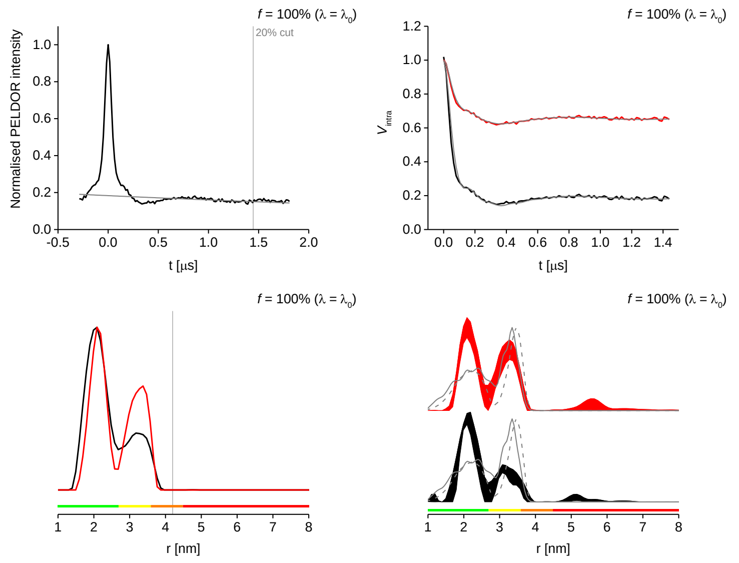

Figure S12: X-band PELDOR data for MscL V120R1, fully labeled, full λ. See page 6 for details.

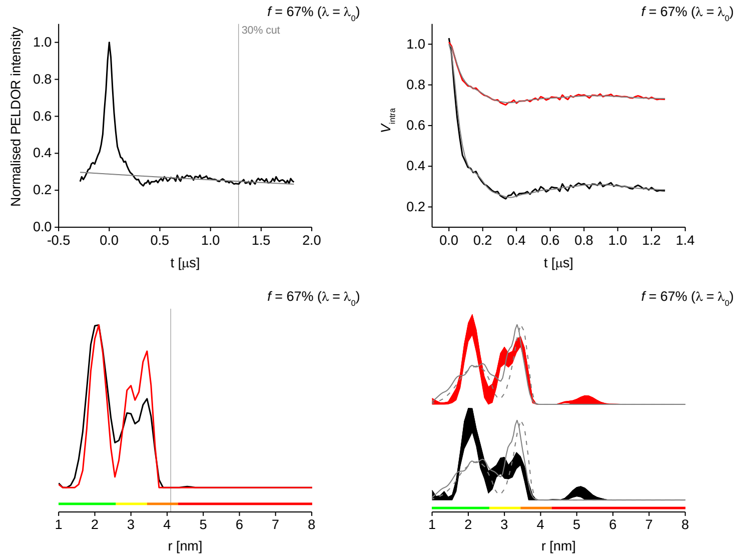

Figure S13: X-band PELDOR data for MscL V120R1 at 67% labeling, full λ. See page 6 for details.

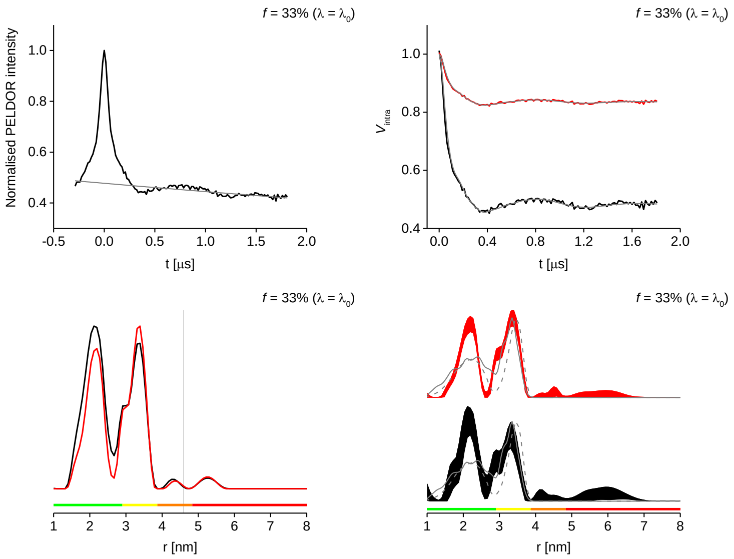

Figure S14: X-band PELDOR data for MscL V120R1 at 33% labeling, full λ. See page 6 for details.

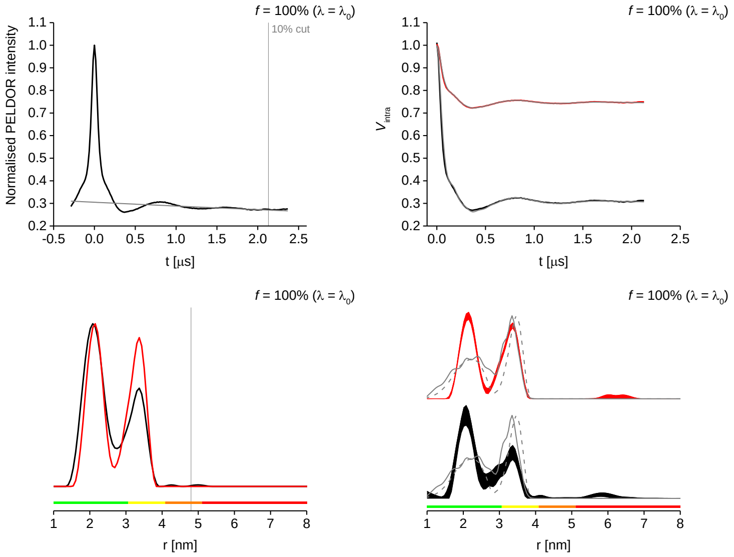

Figure S15: Q-band PELDOR data for MscL V120R1 at 100% labeling, full λ. See page 6 for details.

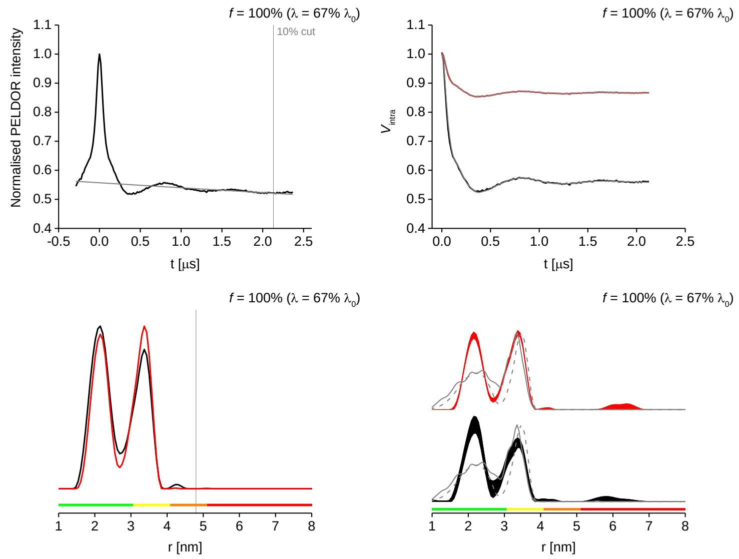

Figure S16: Q-band PELDOR data for MscL V120R1 at 100% labeling, λ = 67% λ_0. See page 6 for details.

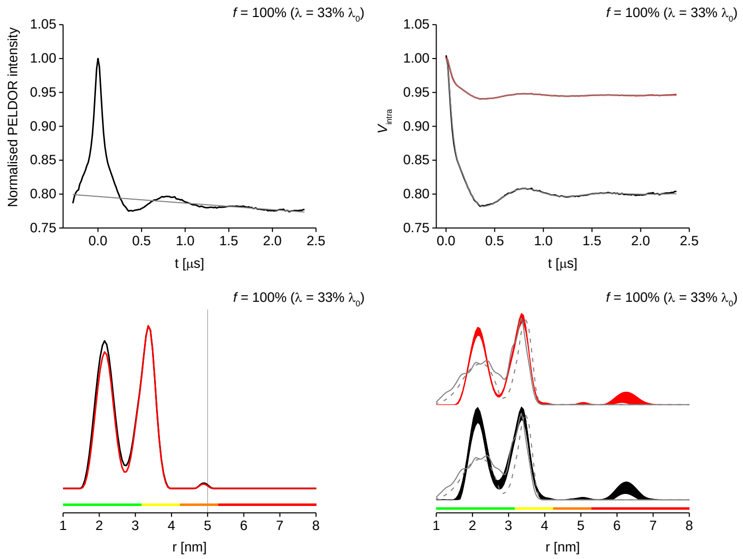

Figure S17: Q-band PELDOR data for MscL V120R1 at 100% labeling, λ = 33% λ_0. See page 6 for details.

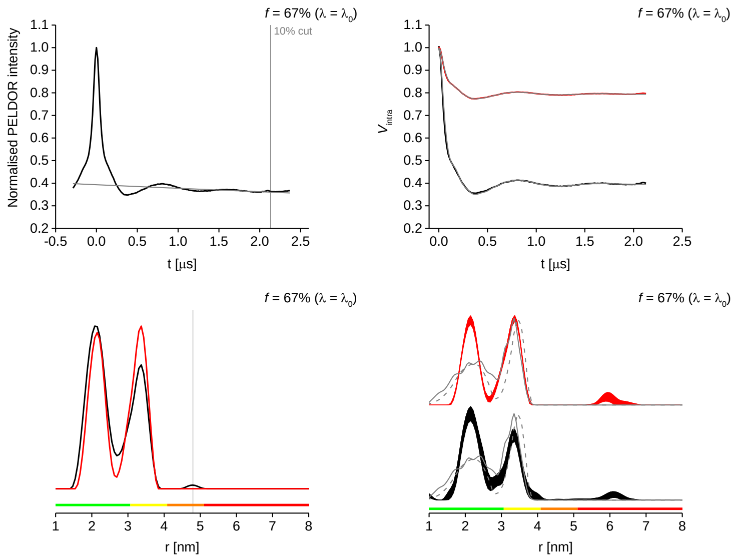

Figure S18: Q-band PELDOR data for MscL V120R1 at 67% labeling, full λ. See page 6 for details.

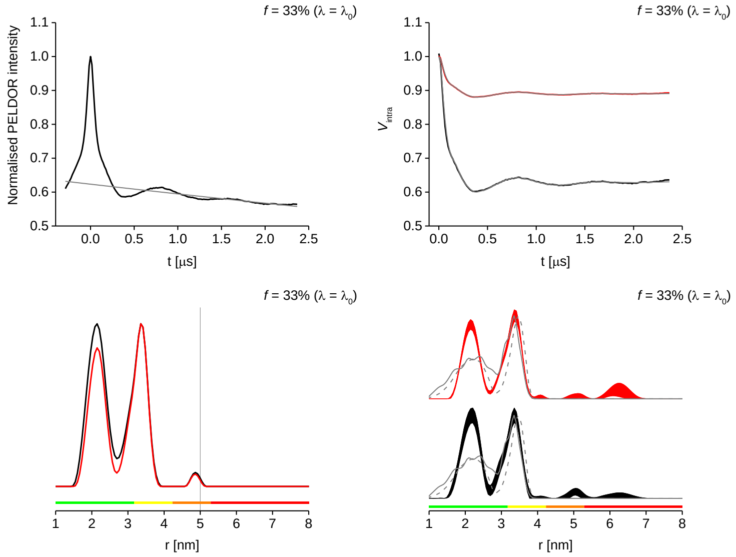

Figure S19: Q-band PELDOR data for MscL V120R1 at 33% labeling, full λ. See page 6 for details.

2.3) MscS D67R1 (transmembrane mutant)

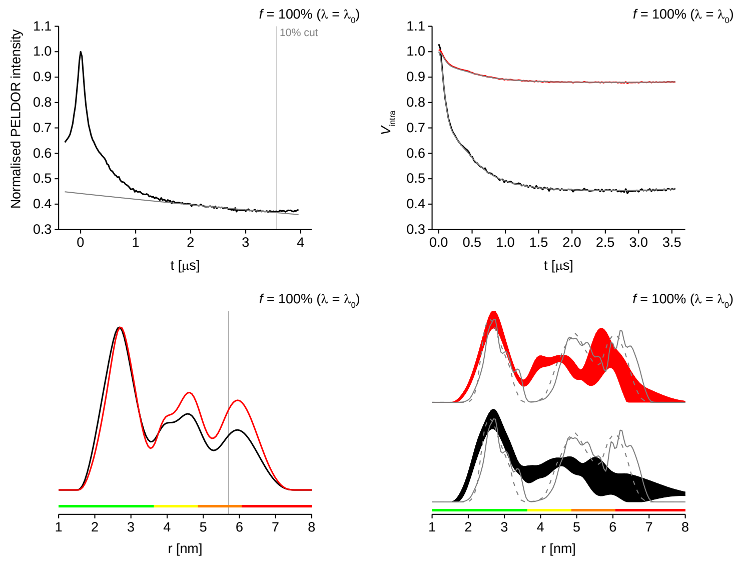

Figure S20: Q-band PELDOR data for MscS D67R1 at 100% labeling, full λ. See page 6 for details.

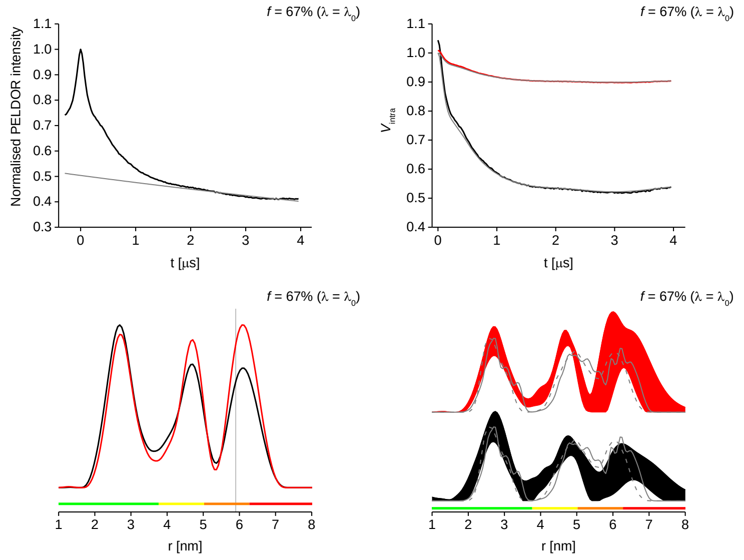

Figure S21: Q-band PELDOR data for MscS D67R1 at 67% labeling, full λ. See page 6 for details.

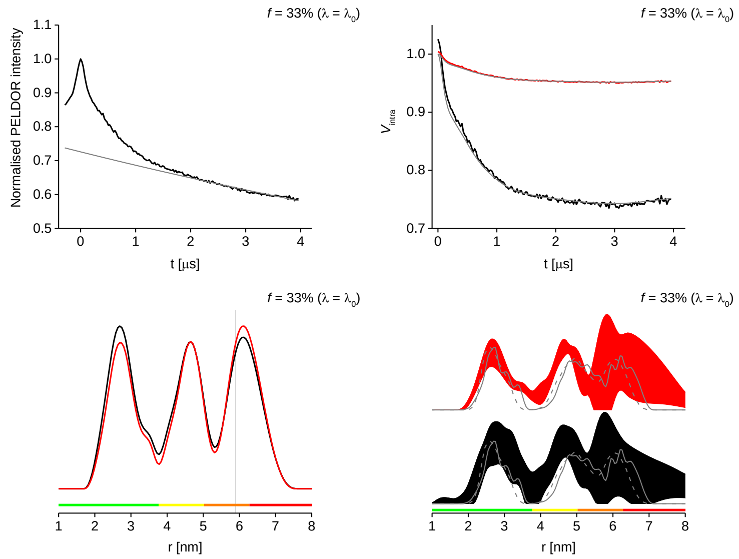

Figure S22: Q-band PELDOR data for MscS D67R1 at 33% labeling, full λ. See page 6 for details.

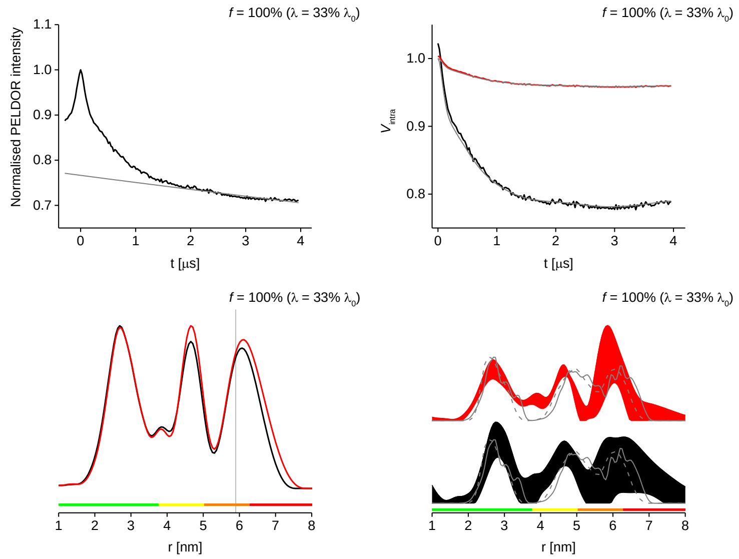

Figure S23: Q-band PELDOR data for MscS D67R1 at 100% labeling, λ = 33% λ_0. See page 6 for details.

2.4) MscL M94R1 (transmembrane mutant)

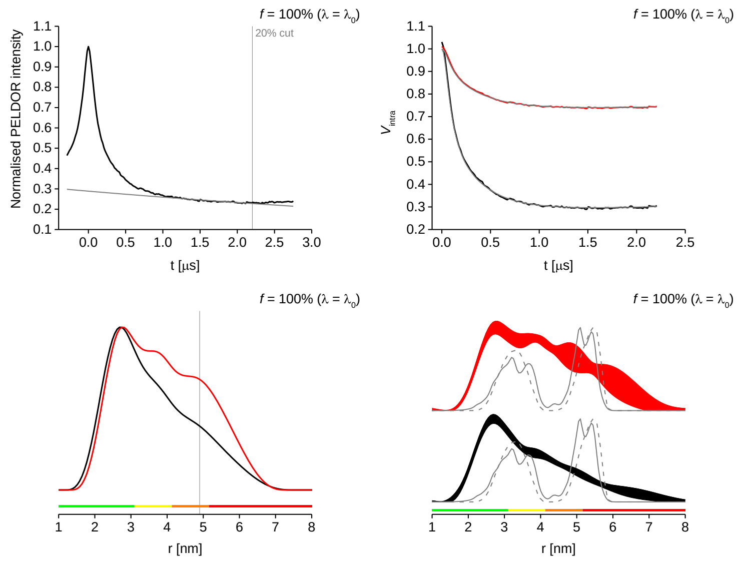

Figure S24: Q-band PELDOR data for MscL M94R1 at 100% labeling, full λ. See page 6 for details.

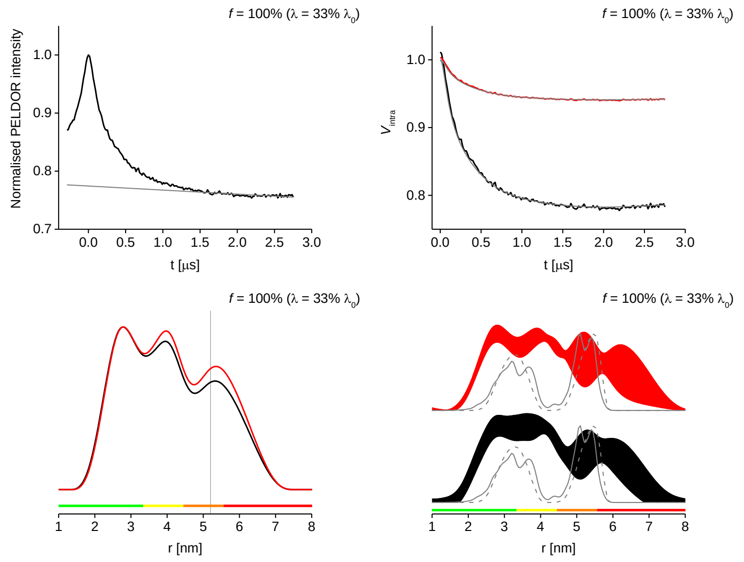

Figure S25: Q-band PELDOR data for MscL M94R1 at 100% labeling, $\lambda = 33\% \lambda_0$. See page 6 for details.

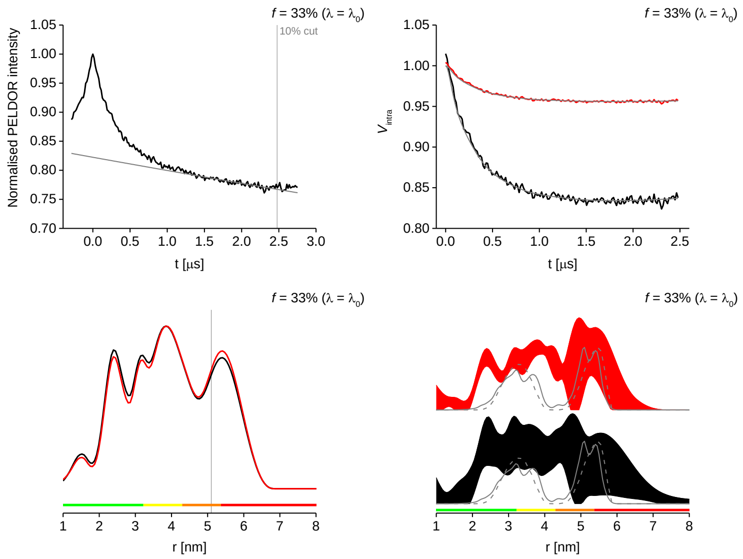

Figure S26: Q-band PELDOR data for MscL M94R1 at 33% labeling, full λ. See page 6 for details.

MscL M94 R1 – second data set with longer *t* (4 µs) used only for scaled fits (see below)

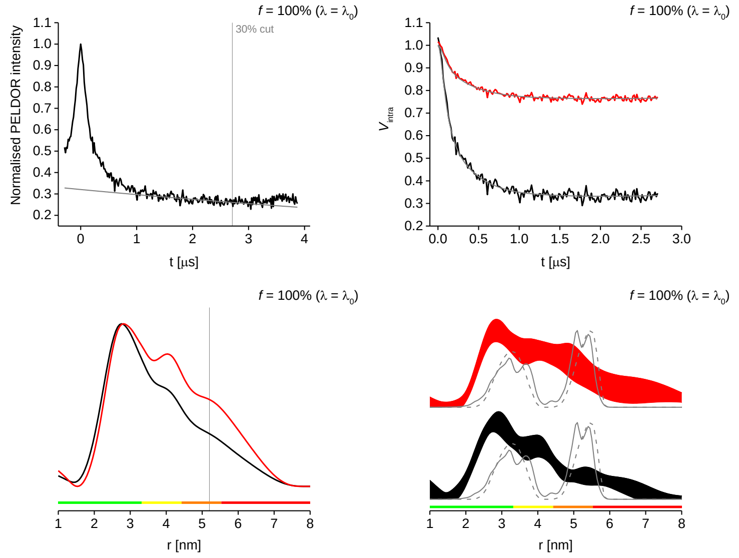

Figure S27: Q-band PELDOR data for MscL M94R1 at 100% labeling, full λ. See page 6 for details.

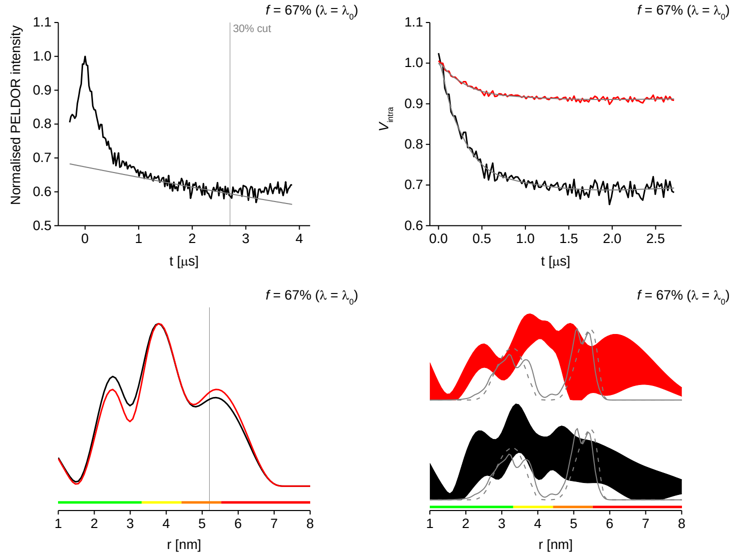

Figure S28: Q-band PELDOR data for MscL M94R1 at 67% labeling, full λ. See page 6 for details.

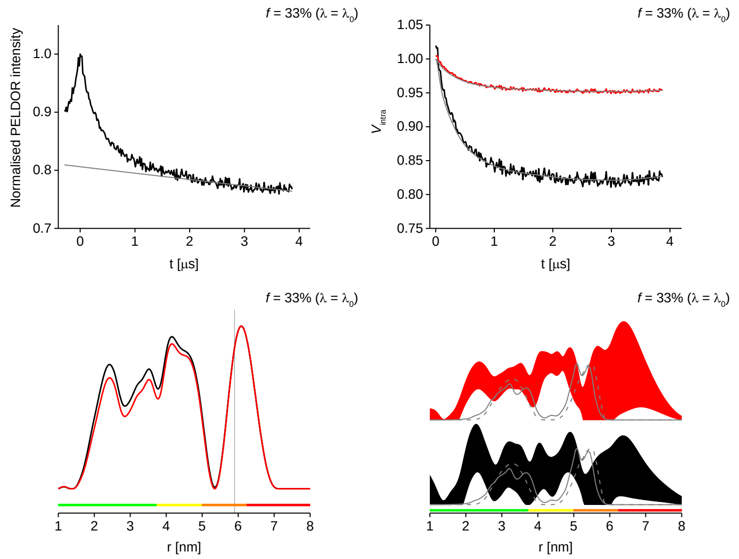

Figure S29: Q-band PELDOR data for MscL M94R1 at 33% labeling, full λ. See page 6 for details.

MscL M94 R1 – scaled fits

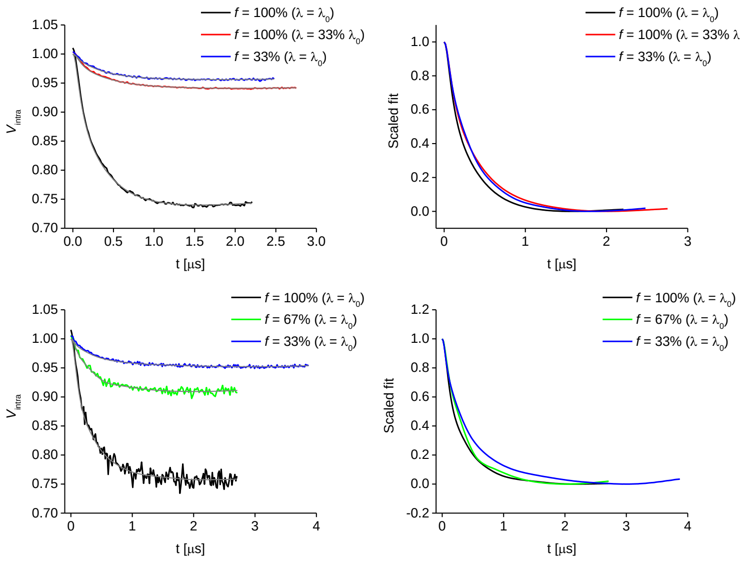

Figure S30: Background-corrected traces (left) and scaled fits (right) for MscL M94R1. Data shown in the top row corresponds to the PELDOR distance distributions shown in the manuscript; detailed PELDOR data for the bottom row are given above. The dipolar evolution functions illustrate the slightly reduced decay of the intramolecular signal observable as a 'reduced curvature' with reduced labeling and λ.

Chapter 3: Dipolar dephasing

The contribution of dipolar dephasing to the signal decay was tested by means of Hahn echo decays varying the flip-angle of the second pulse from π to π/5. A pure instantaneous diffusion effect would be expected to follow Eq. S1:

$$\frac{V(2\tau)}{V(0)} = exp\left[- \frac{2\pi}{9\times\sqrt{3}} \frac{\mu_0 g^2 \beta_e^2}{\hbar} C\tau \int sin^2\left(\frac{\beta(\Omega_S)}{2}\right) f(\Omega_S) d\Omega_S\right] \qquad \text{Equation S1}$$

g is the proportionality factor (g-value), $\hbar$ the Planck constant divided by 2π, μ_0 the permeability of the vacuum, τ the interpulse delay, C the volume concentration of the spins, $\beta(\Omega_S)$ the flip angle of the second pulse as a function of resonance offset, Ω_S the resonance offset and $f(\Omega_S)$ the EPR lineshape function.

According to Eq. S1 (6) the contribution from instantaneous diffusion would reduce to 9.6% of that caused by a π pulse when comparing to that raised by a π/5 pulse which will be considered as having diminished dipolar dephasing.

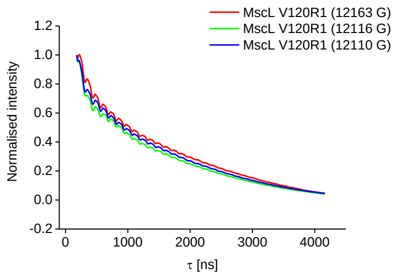

Figure S31: The magnetic field (B) was varied from 12090 to 12210 G, with 12116 G being the field where the signal maximum is found in the field swept EPR spectrum. As expected the higher the spectral intensity the higher the dephasing rate, with the fastest decay being measured at the field of maximum spectral intensity (12116 G).

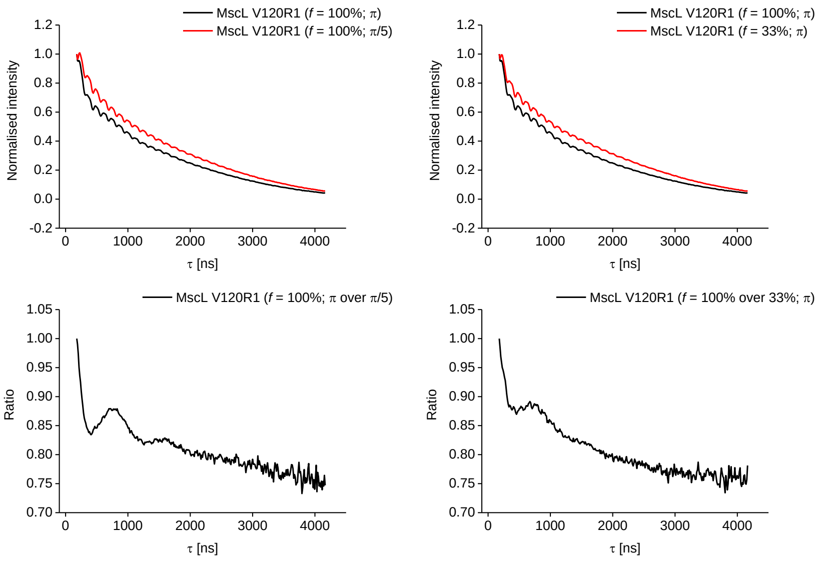

Figure S32: Decay traces from dipolar dephasing experiments on MscL V120R1 for the fully labeled sample (top left) and comparing π decay rates for the 100% vs. the 33% labeled sample (top right). Corresponding ratios are shown in the bottom row.

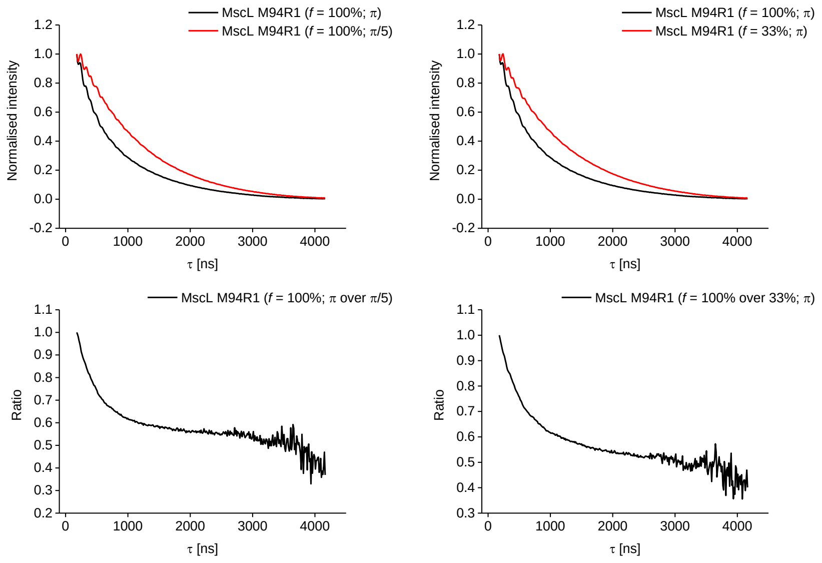

Figure S33: Decay traces from dipolar dephasing experiments on MscL M94R1 for the fully labeled sample (top left) and comparing π decay rates for the 100% vs. the 33% labeled sample (top right). Corresponding ratios are shown in the bottom row.

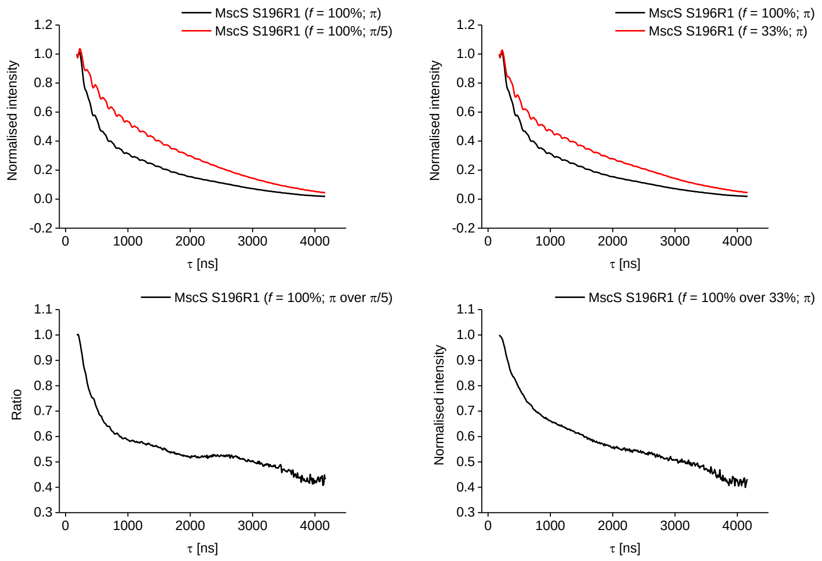

Figure S34: Decay traces from dipolar dephasing experiments on MscS S196R1 for the fully labeled sample (top left) and comparing π decay rates for the 100% vs. the 33% labeled sample (top right). Corresponding ratios are shown in the bottom row.

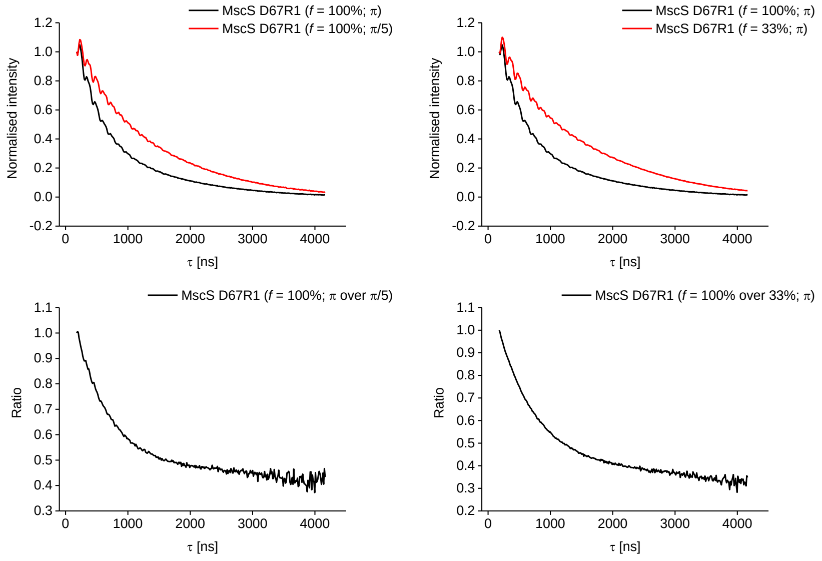

Figure S35: Decay traces from dipolar dephasing experiments on MscS D67R1 for the fully labeled sample (top left) and comparing π decay rates for the 100% vs. the 33% labeled sample (top right). Corresponding ratios are shown in the bottom row.

Chapter 4: Peak intensities

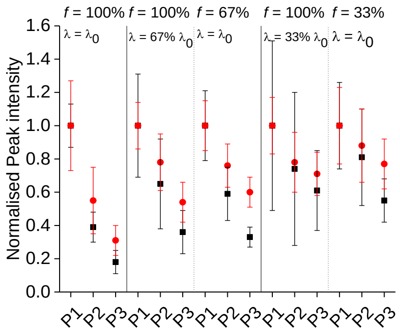

Figure S36: Peak integrals (mean ±2σ confidence intervals) for MscS S196R1. P1 = shortest, P2 = middle, P3 = longest distance peak; black: non-scaled data, red: power-scaled data.

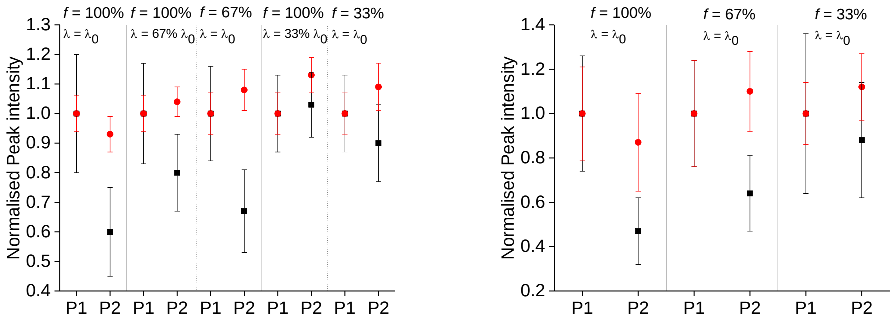

Figure S37: Peak integrals (mean ±2σ confidence intervals) for MscL V120R1. P1 = short, P2 = long distance peak. Left: Q-band; right: X-band; black: non-scaled data, red: power-scaled data.

Chapter 5: Supporting References

1. Krivov, G. G., M. V. Shapovalov, and R. L. Dunbrack, Jr. 2009. Improved prediction of protein side-chain conformations with SCWRL4. Proteins 77:778-795.
2. Polyhach, Y., E. Bordignon, and G. Jeschke. 2011. Rotamer libraries of spin labelled cysteines for protein studies. Phys. Chem. Chem. Phys. 13:2356-2366.
3. Hagelueken, G., R. Ward, J. H. Naismith , O. Schiemann. 2012. MtsslWizard: In Silico Spin-Labeling and Generation of Distance Distributions in PyMOL. Appl. Magn. Reson. 42:377-391.
4. Perozo, E., A. Kloda, D. M. Cortes , B. Martinac. 2001. Site-directed spin-labeling analysis of reconstituted Mscl in the closed state. J. Gen. Physiol. 118:193-206.
5. Jeschke, G., V. Chechik, P. Ionita, A. Godt, H. Zimmermann, J. Banham, C. R. Timmel, D. Hilger , H. Jung. 2006. DeerAnalysis2006 - a comprehensive software package for analyzing pulsed ELDOR data. Appl. Magn. Reson. 30:473-498.
6. Schweiger, A., and G. Jeschke. 2001. Principles of pulse electron paramagnetic resonance. OUP Oxford, New York.
